# Pharmacophylogenetic relationships of genus *Dracocephalum* and its related genera based on multifaceted analysis

**DOI:** 10.3389/fphar.2024.1449426

**Published:** 2024-10-03

**Authors:** Haolin Liu, Xiaowei Feng, Yulian Zhao, Guoshuai Lv, Chunhong Zhang, Aruhan, Tsend-Ayush Damba, Na Zhang, Dacheng Hao, Minhui Li

**Affiliations:** ^1^ College of Pharmacy, Inner Mongolia Medical University, Hohhot, Inner Mongolia, China; ^2^ Department of Pharmacy, Baotou Medical College, Baotou, Inner Mongolia, China; ^3^ Central laboratory, Inner Mongolia Autonomous Region Hospital of Traditional Chinese Medicine, Hohhot, Inner Mongolia, China; ^4^ Department of Mongolia Medicine Study, Institute of Traditional Medicine and Technology of Mongolia, Ulaanbaatar, Mongolia; ^5^ Liaoning Provincial Universities Key Laboratory of Environmental Science and Technology, School of Environment and Chemical Engineering, Dalian Jiaotong University, Dalian, China

**Keywords:** *Dracocephalum*, pharmacophylogeny, geographical distribution, plant metabolites, pharmacological activities

## Abstract

The Lamiaceae genus *Dracocephalum*, with over 30 species, is believed to have considerable medicinal properties and is widely used in Eurasian ethnomedicine. Numerous studies have researched on the geographical distribution, metabolite identification, and bioactivity of *Dracocephalum* species, especially amidst debates concerning the taxonomy of its closely related genera *Hyssopus* and *Lallemantia*. These discussions present an opportunity for pharmacophylogenetic studies of these medicinal plants. In this review, we collated extensive literature and data to present a multifaceted view of the geographical distribution, phylogenetics, phytometabolites and chemodiversity, ethnopharmacological uses, and pharmacological activities of *Dracocephalum*, *Hyssopus*, and *Lallemantia*. We found that these genera were concentrated in Europe, with species adapted to various climatic zones. These genera shared close phylogenetic relationships, with *Dracocephalum* and *Hyssopus* displaying intertwined patterns in the phylogenetic tree. Our review assessed more than 900 metabolites from these three genera, with terpenoids and flavonoids being the most abundant. Researchers have recently identified novel metabolites within *Dracocephalum*, expanding our understanding of its chemical constituents. Ethnopharmacologically, these genera have been traditionally used for treating respiratory, liver and gall bladder diseases. Extracts and metabolites from these genera exhibit a range of pharmacological activities such as hepatoprotective, anti-inflammation, antimicrobial action, anti-hyperlipidaemia, and anti-tumour properties. By integrating phylogenetic analyses with network pharmacology, we explored the intrinsic links between metabolite profiles, traditional efficacy, and modern pharmacology of *Dracocephalum* and its related genera. This study contributes to the discovery of potential medicinal value from closely related species of *Dracocephalum* and aids in the development and sustainable use of medicinal plant resources.

## 1 Introduction

The Lamiaceae family is the sixth most diverse in terms of species and the tenth most diverse in terms of genera among angiosperms (Christenhusz and Byng, 2016). With its cosmopolitan distribution, this botanical group is used extensively in various fields. For example, lavender [Lamiaceae; *Lavandula angustifolia* Mill.], which is known for its ornamental cultivation and aromatic qualities, has attracted considerable scholarly attention (Gu et al., 2021). Another member of this family, fresh mint [Lamiaceae; *Mentha piperita* L.], is highly valued for its culinary use (Hsu et al., 2010). Historical records from various regions and ethnic groups have confirmed the traditional medicinal uses of Lamiaceae species. In Traditional Chinese Medicine, the dried roots and rhizomes of *Salvia miltiorrhiza* Bunge [Lamiaceae; Salviae miltiorrhizae radix et rhizoma] is lauded for its therapeutic properties, which encompass the promotion of blood circulation, the alleviation of menstrual discomfort, heat-clearing, and the reduction of swelling (Deng et al., 2016). In the southern regions of India, *Leucas ciliata* Benth [Lamiaceae] have been documented for their efficacy in wound healing and as a snakebites remedy (Akshatha et al., 2021). Additionally, the native American plant *Callicarpa americana* L [Lamiaceae] has been traditionally used by several indigenous tribes in the southeastern United States for its febrifuge, stomachic, and anti-dysenteric properties (Dettweiler et al., 2020).

**FIGURE 1:**
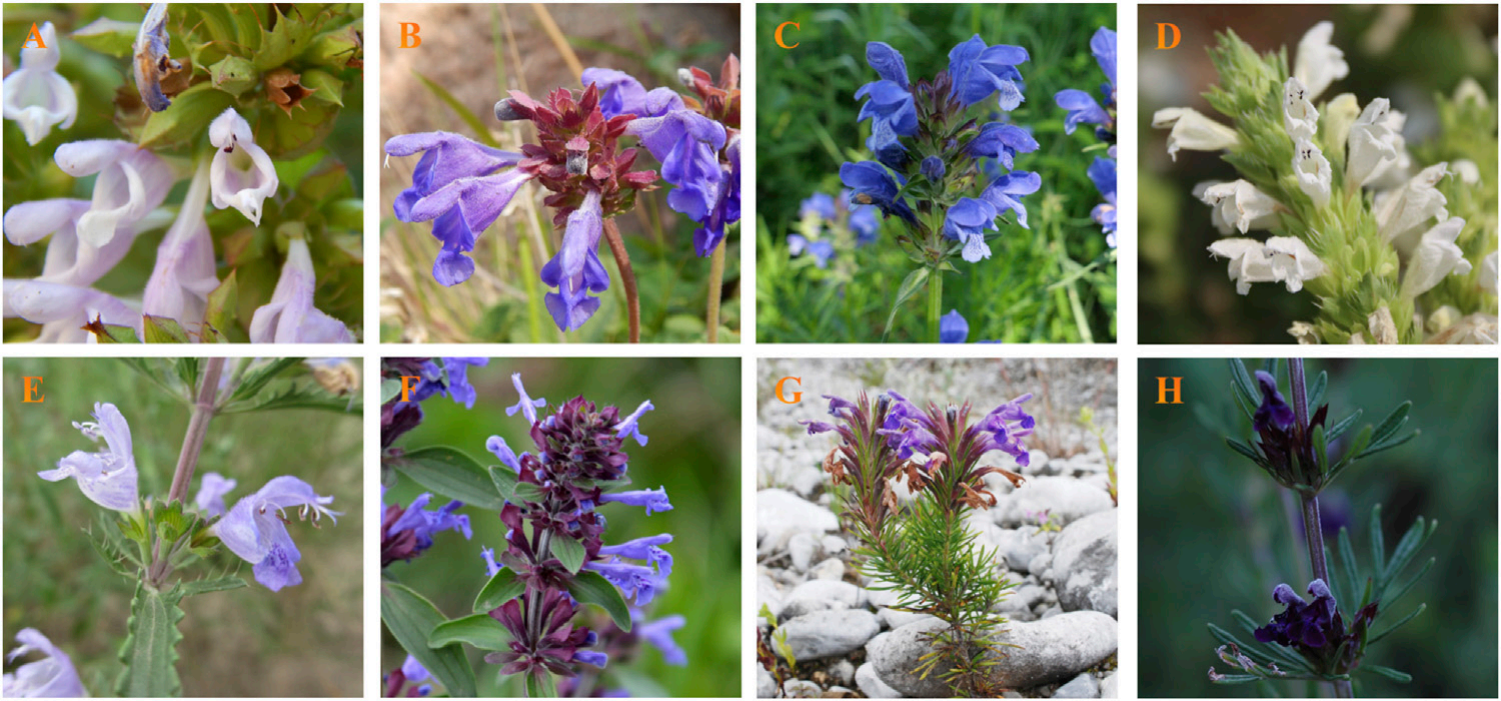
Species within the *Dracocephalum* genus, recognized for their medicinal properties, display certain distinctive features in the field of plant morphology. *D. rigidulum* **(A)**, *D. rupestre* **(B)**, *D. argunense* **(C)**, *D. heterophyllum* **(D)**, *D. moldavica* **(E)**, **(D)** *nutan*s **(F)**, *D. forrestii* **(G)**, *D. taliense* **(H)**. The picture comes from shooting.

Globally, the importance of Lamiaceae plants is undeniably prominent. The genera *Dracocephalum*, *Hyssopus*, and *Lallemantia* belong to the Lamiaceae family. In recent years, the phylogenetic relationships among these three genera have attracted the attention of researchers and their taxonomy has been reassessed from multiple perspectives, including bioinformatics, geography, and plant morphology. Among them, *Dracocephalum* has the greatest number of species, characterized by flowers that are typically blue-purple with occasional whites, and bracts, that are often obovate and frequently feature acute teeth or spines, rarely being entire. The floral structure is described as tubular or campanulate-tubular, either straight or slightly curved, and adorned with 15 veins and five teeth (Figure 1). As close relatives of the *Dracocephalum* genus, the flowers of *Hyssopus* and *Lallemantia* genera also typically have a tubular calyx, 15 veins, five teeth, and a bilabiate corolla, with four stamens and a stigma that is bifid at the apex. However, significant differences between the stems and leaves were observed. Plants of these three genera contain several valuable metabolites. Notably, *Dracocephalum* is known for its rich content of essential oils, flavonoids, glycosides, triterpenoids, organic acids, and esters (Lv et al., 2022). The ‘A Quick-Consultative Dictionary of World Medicinal Plants’ documents the nomenclature, medicinal parts, habitats, and therapeutic properties of the medicinal plants within the *Dracocephalum* genus. A substantial proportion of these records are from diverse provinces across China, where they have garnered attention for their roles in traditional Chinese medicine, particularly for their heat-clearing, blood-cooling, and cough-relieving effects (Jiang, 2015; Yao et al., 2020; Yan et al., 2020).

As living standards progressively increase, societal emphasis on health and wellbeing increases. This trend is mirrored by an increasing interest in herbal resources. However, the growing demand for herbal resources could result in the overexploitation of wild resources and a shortage of medicinal plants that are on the verge of extinction. Thus, sustainable use of medicinal plants has become a pivotal research domain in contemporary society. This field demands a robust foundation of precise knowledge and strategic application of theoretical frameworks, as exemplified by the discipline of pharmacophylogeny. The concept of pharmacophylogeny was proposed by Xiao Peigen in the 1980s and is an emerging discipline that studies the correlation between the molecular phylogeny of medicinal plants, chemical metabolites, pharmacological activities, and traditional applications. Pharmacophylogeny is advantageous owing to its interdisciplinarity and interpenetration feature its research objects, which involve multidisciplinary fields (Chen et al., 2005; Hao and Xiao, 2020). Establishing this subject as an essential guide for the conservation and development of medicinal plant resources is crucial, and justified through long-term research (Gong et al., 2022; Hao et al., 2022). Pharmacophylogeny proposes that phylogenetically closer taxonomic groups, such as families, tribes, or genera, are more likely to possess similar chemical profiles, which contribute to more analogous bioactivities. Pharmacophylogeny principles have been applied to various aspects of imported drug resource substitution, the search for new drugs, and production practices to expand the development and utilization of medicinal resources (Li, 2021; Hao et al., 2022).

In recent years, many studies have been conducted on the genus *Dracocephalum*, covering various aspects such as the geographical distribution of its species, their phylogenetic relationships with other Lamiaceae genera, its metabolites, and its pharmacological activities. Our review meticulously compiled extensive information on *Dracocephalum* species and their closely related genera to clarify the current status of *Dracocephalum* as a medicinal plant through the lens of pharmacophylogenetic principles. This compendium is intended not only to deepen our understanding of *Dracocephalum* and its relatives but also to encourage the sustainable management and application of these precious botanical resources.

For this review, we have primarily referenced ‘A Quick-Consultative Dictionary of World Medicinal Plants’ and conducted independent searches for the species names across PubMed (https://pubmed.ncbi.nlm.nih.gov/), Google Scholar (https://scholar.google.com), and the China National Knowledge Infrastructure (https://kns.cnki.net/) database, focusing on the most current and authoritative literature. All plant names mentioned in this article have been confirmed on the WFO Plant List (https://wfoplantlist.org/), ensuring that they are accepted species names. The geographical coordinates were sourced from the Global Biodiversity Information Facility (https://www.gbif.org/zh/). Species distribution data were integrated from the WFO, WORLD PLANTS (https://www.worldplants.de/), and iPlant (http://www.iplant.cn/) databases, facilitating the delineation of climatic conditions. Phylogenetic tree sequences are derived from the National Center for Biotechnology Information (NCBI) (https://www.ncbi.nlm.nih.gov/) and the scholarly work of Chen G. W. et al. (2022).

## 2 Geographical distribution and phylogenetics

### 2.1 Geographical distribution

The genus *Dracocephalum*, comprising over 60 species, is distributed across the Eurasian and North American tectonic plates. The speciation of *Dracocephalum* exhibits regional differentiation, featuring four primary distribution areas on the Eurasian plate and one main area on the Indian and American plates. The genus *Dracocephalum* exhibits an endemic distribution, with robust its adaptability observed predominantly within the climatic zones of the Northern Hemisphere, specifically in temperate continental climate areas.

In the Asian continent, the genus *Dracocephalum* shows a rich diversity of species that exhibit distinct climatic differentiation and a geographical distribution gradient from east to west. *Dracocephalum’s* distribution transitions from temperate zones to more demanding alpine mountainous regions, with this genus predominantly thriving in temperate continental climates. These climates are marked by significant seasonal variations, pronounced temperature fluctuations, and moderate, seasonally concentrated rainfall, which, along with the region’s topography, shape the ecological spread of *Dracocephalum* across alpine, temperate monsoon, and Mediterranean climates.

The aridification of the Asian interior and the uplift of the Qinghai-Tibet Plateau (QTP) are substantial geological and climatic events that have influenced the speciation and distribution of *Dracocephalum*. Interior aridification likely drove the rapid radiation of the genus, whereas the uplift of the QTP initiated its dispersal and diversification in the plateau and surrounding areas (Chen Y. P. et al., 2022). The flora of Central and West Asia are closely related to the QTP, with some species hypothesized to have originated from the plateau and later migrated to other regions (Wen et al., 2014; Zhang et al., 2017). The alpine climate of the QTP, influenced by its distance from the ocean, high terrain, and low temperatures, has fostered a conducive environment for the growth and diversification of this genus. Interestingly, despite the southern part of the American Plate fallings within the temperate continental climate zone, there are no records of *Dracocephalum* in this region, as the genus’s adaptive radiation of this genus may be unable to cross the tropical climate zones of the American Plate.

**FIGURE 2:**
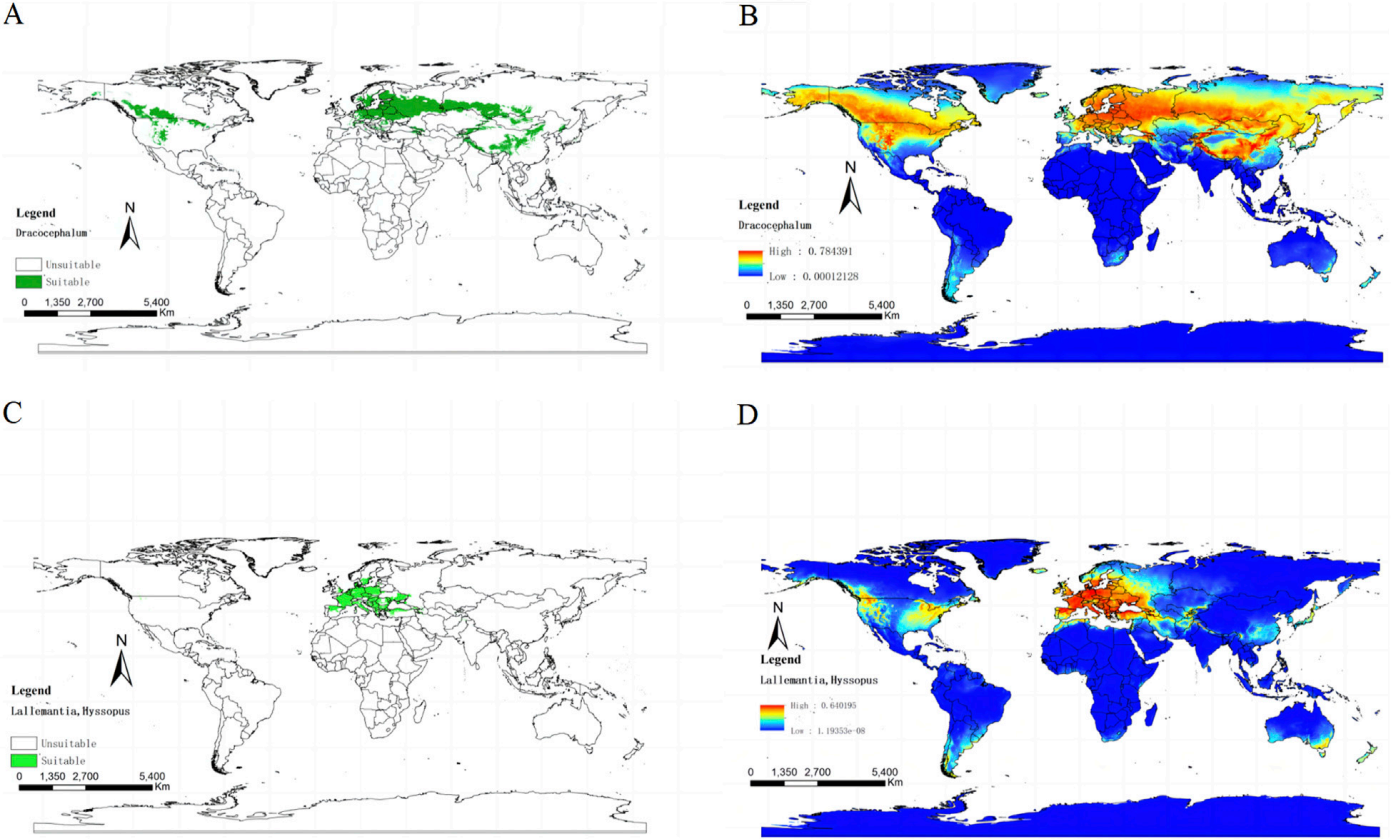
Ecologically suitable regions of genus *Dracocephalum* (Green distribution is suitable for the growth area of *Dracocephalum*). **(A)**. Suitability distribution of *Dracocephalum*. From blue to red, the suitability of *Dracocephalum* gradually increases, and yellow is the transition area **(B)**. Ecologically suitable regions of genus *Lallemantia* and *Hyssopus* (Green distribution is suitable for the growth area of *Lallemantia* and *Hyssopus*). **(C)**. Suitability distribution of *Lallemantia* and *Hyssopus*. From blue to red, the suitability of *Lallemantia* and *Hyssopus* gradually increases, and yellow is the transition area **(D)**.

The spatial distribution pattern (Figures 2A, C) shows that *Dracocephalum* has a broader distribution than the *Lallemantia* and *Hyssopus*; however, the latter three genera share overlapping ranges within Europe. *Dracocephalum* is found across Asia and Europe, whereas *Lallemantia* and *Hyssopus* are predominantly present in Southern European countries such as Greece and Italy, in addition to other regions across Europe. The distribution of *Dracocephalum* also extends to the Americas, particularlynorthern Mexico. From the perspective of suitable ecological zones, the genera *Dracocephalum*, *Lallemantia*, and *Hyssopus* are well adapted across much of Europe. *Dracocephalum*, in particular, is amenable to cultivation and thrives along the Mediterranean coast, Central Asia, West Asia, and the western regions of North America. However, most areas south of the equator were not conducive to the cultivation and growth of these three genera (Figures 2B, D).

### 2.2 Phylogenetics

The genus *Dracocephalum* encompasses a diverse array of over 60 species, approximately 30 of which are recognized for their medicinal properties. The majority of these medicinal species are meticulously documented in the authoritative publication ‘A Quick-Consultative Dictionary of World Medicinal Plants’, as well as in the traditional medical literature of numerous ethnic groups and within a variety of scholarly texts. Currently, phylogenetic analysis is widely applied in pharmacophylogeny, especially in the exploration and organization of traditional Chinese medicine (Hao et al., 2017; Gong et al., 2022). The medicinal plants of *Dracocephalum* are underpinned by a rich and extensive genomic sequence foundation, which renders them invaluable subjects for extensive and scholarly research.

In 2022, Chen et al. conducted an exhaustive study employing a range of genetic sequences, including chloroplast sequences (rpl32-trnL, trnL-trnF, ycf1, ycf1-rps15), nuclear genes (ITS and ETS), and low-copy nuclear gene sequences (AT3G09060, AT1G09680) (Chen Y. P. et al., 2022). Despite the topological structures of the resultant phylogenetic trees demonstrating variability, these findings suggest that the taxonomic classification of *Dracocephalum* species is a matter of ongoing debate. This is particularly true when considering the phylogenetic interrelationships among *Hyssopus*, *Lallemantia*, and *Dracocephalum*, which present both morphological and taxonomic challenges.

**FIGURE 3:**
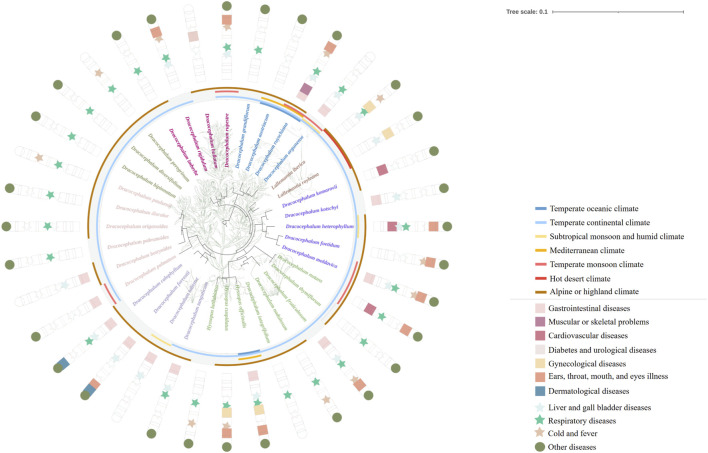
This refined phylogenetic tree expands upon the original framework constructed by Chen et al., with selective pruning to emphasize species of Dracocephalum and its closely related genera, recognized for their medicinal properties and historical use in healing practices. The outermost ring of the visualisation encapsulates the climatic distribution characteristics of these plants, collated from the World Plants database [https://www.worldplants.de/world-plants-complete-list/complete-plant-list] and the iPlant database [http://www.iplant.cn/]. The annotations on the outer layer provide insights into the medicinal efficacy attributed to these species, offering a visual representation of their therapeutic potential. The traditional classification of efficacy is shown in Supplementary Table S2, and the climate information of each region is shown in Supplementary Table S5.

The ITS sequence, which is a standard in pharmacophylogeny analysis, was pivotal in our study (Hao et al., 2017; Wang et al., 2019). We used the Neighbor-Joining (NJ) model to construct phylogenetic trees (Supplementary Figure S1) from these sequences (Supplementary Table S1). These result indicates that within the medicinal flora of *Dracocephalum* and its closest botanical allies, *Hyssopus* is consistently intermingled with the *Dracocephalum* lineage, whereas *Lallemantia* may be more appropriately classified taxonomically as a distinct entity. However, it is important to note that the phylogenetic branches derived from this model had relatively low bootstrap values. In a stark comparison, Chen G. W. et al. (2022) leveraged the Pentapeptide Repeat (PPR) motif and applied the Bayesian Inference (BI) algorithm to uncover a tree with significantly robust branch support. By extracting the topological structure of the medicinal plant branches from their analysis, we delineated a novel phylogenetic tree for the genus *Dracocephalum* and its related medicinal species (Figure 3). Within the *Dracocephalum* genus, species such as *Dracocephalum argunense* Fisch. ex Rchb. and *Dracocephalum bipinnatum* Rupr., demonstrated adaptability to two diverse climatic conditions. It is worth noting that, *Dracocephalum moldavica* L. and *Dracocephalum nutans* L. have the ability to inhabit three separate climatic zones. Furthermore, *Dracocephalum ruyschiana* L. had an even broader distribution across the five climatic regions. In contrast to medicinal plants in the genera *Hyssopus* and *Lallemantia*, which share a close phylogenetic affinity with *Dracocephalum*, only *Hyssopus officinalis* L. and *Lallemantia royleana* (Benth.) Benth. are documented to have distributions spanning multiple climatic zones. This adaptability highlights the ecological plasticity of these species and underscores their potential for medicinal use in various environmental contexts.

## 3 Phytometabolites and chemodiversity

**FIGURE 4:**
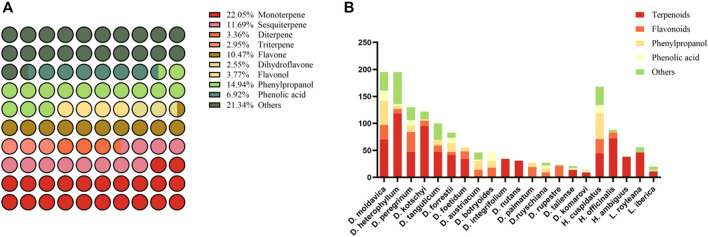
A proportional diagram of all metabolites collected from the medicinal plants of the *Dracocephalum* genus and its closely related genera **(A)**, and information on each collected medicinal plant metabolite **(B)**.

Our survey systematically catalogued the chemical metabolites from a total of seventeen *Dracocephalum* species, three *Hyssopus* species, and two *Lallemantia* species, resulting in the identification of more than 900 metabolites (Supplementary Tables S3, S4). The predominant metabolites are terpenoids, flavonoids, phenylpropanoids, and phenolic acids. Fatty acids, alkaloids, and steroids, being less prevalent, were categorized under the ‘Others’ grouping (as depicted in Figure 4A). Our findings, which only provide a consolidated summary, consider the variability in extraction methods reported in the literature. Notably, within the genus *Dracocephalum*, *D. moldavica* exhibited the greatest metabolite diversity, while in *Hyssopus*, *Hyssopus cuspidatus* Boriss. had the most characterized metabolites. In the genus *Lallemantia*, the number of reported metabolites for *L. royleana* significantly surpassed that for *Lallemantia iberica* (M.Bieb.) Fisch. & C.A.Mey., with a focus on the aerial parts of the plants rather than on the seeds (Figure 4B). Most studies have focused on identifying chemical metabolites in naturally dried whole plants of *Dracocephalum* species, with *D. moldavica*, *Dracocephalum heterophyllum* Benth., and *D. nutans* being the most studied examples (Jiang, 2015). Some studies have specifically analyzed whole plants during the flowering stage, such as *Dracocephalum kotschyi* Boiss., *Dracocephalum palmatum* Stephan ex Willd., *Dracocephalum austriacum* L. and *Dracocephalum botryoides* Steven, which may correspond to the peak times of traditional medicinal activity (Olennikov et al., 2017; Kashchenko et al., 2022). Additionally, some investigations have concentrated on particular plant organs, such as leaves or roots (Poursalavati et al., 2021; Kashchenko et al., 2022). Concurrently, many novel metabolites are continually being discovered within this flora, highlighting the dynamic chemical landscape of this genus. This disparity may be attributed to variations in metabolic pathways, ecological preferences, or evolutionary factors that have shaped the chemical diversity of these plants. It is important to acknowledge that current research on the metabolites of *Dracocephalum* and its closely related genera are not exhaustive, indicating the need for further comprehensive studies. However, the extensive geographical distribution of *Dracocephalum* species, along with the significant altitudinal gradients they inhabit, make them an excellent model for studying the relationship between medicinal plant metabolites and environmental factors such as latitude, longitude, and altitude. This research is crucial for understanding the influence of environmental factors on the chemical profiles of medicinal plants and has implications for biodiversity conservation and the discovery of new pharmaceutical metabolites.

In this review, we focused on terpenoids and flavonoids, which constitute the two most diverse classes of metabolites in the aforementioned genera. Moreover, during our literature review, we found that researchers were often able to successfully isolate and characterize new metabolites from the genus *Dracocephalum*. Among these, some have already made initial contributions to the advancement of human health, whereas others are yet to be investigated because of their unknown activities.

### 3.1 Terpenoids

Terpenoids are ubiquitous throughout the plant kingdom and play integral roles in both primary and secondary metabolic processes. They are crucial for a myriad of biological functions, such as signal transduction, reproduction, communication, and adaptation of plants to environmental changes, including climate change, as well as their defense mechanisms (Tholl, 2015; Atriya et al., 2023). Terpenoid metabolites within plants can attract pollinating insects and deter or resist predators and other organisms that may harm the plant foliage. Plants use the color and/or aroma of these volatile metabolites to entice pollinators and animals, thereby facilitating seed dispersal (Boncan et al., 2020; Tetali, 2019). Certain terpenoids not only regulate plant growth and development and respond to environmental pressures but also play a central role in the physiological activities of plants (Chen et al., 2020). Additionally, the majority of plant terpenoids possess antimicrobial and insecticidal properties (Yamaguchi, 2022).

In this study, we compiled a collection of 283 terpenoids from the *Dracocephalum* genus, including 216 monoterpenes, 112 sesquiterpenes, 24 diterpenes, and 29 triterpenes. This collection highlights the rich chemical diversity of this class of metabolites and their potential significance in biological and ecological contexts.

#### 3.1.1 Monoterpenes and sesquiterpenes

Monoterpenes and sesquiterpenes are prevalent in the essential oils of plants and represent the most abundant and varied classes within the genera *Dracocephalum*, *Hyssopus*, and *Lallemantia*. In this literature review, with the exception of essential oils from *Dracocephalum tanguticum* Maxim. extracted via supercritical carbon dioxide, which had a combined GC/MS-determined proportion of monoterpenes, oxygenated monoterpenes, sesquiterpenes, and oxygenated sesquiterpenes of less than 70% (Zhang et al., 2007), species such as *D. heterophyllum*, *D. moldavica*, and *D. nutans* all surpassed this percentage threshold (Zhang et al., 2008; Fattahi et al., 2021; Ashrafi et al., 2017). GC-MS analysis of *Dracocephalum integrifolium* Bunge from various regions revealed that these four metabolite classes comprised an average of 95.86% of the total essential oil content (Zhou et al., 2019). Some of these metabolites exhibited toxicity, suggesting their potential for weed management and resistance to pests and pathogens, thereby warranting consideration for the development of novel natural pesticides.

Specific metabolites, such as citral and nerolidol, which are the most abundant in the essential oil of *D. kotschyi*, may also account for its notable *in vitro* antibacterial activity against gram-negative bacteria (Chahardoli et al., 2019). Among *Hyssopus*, *Lallemantia*, and *Dracocephalum*, there was certain overlap in the presence of monoterpene and sesquiterpene metabolites. Furthermore, some metabolites have been identified that are distinguished solely by their optical isomers within these genera. However, the diversity of these shared metabolites is generally less pronounced in *Hyssopus* and *Lallemantia* than in *Dracocephalum*. This distinction underscores the rich chemical landscape of *Dracocephalum*, which may provide a more extensive reservoir of bioactive metabolites for future research and medicinal exploitation.

#### 3.1.2 Diterpenes and triterpenes

The variety of diterpenes and triterpenes detected in *Dracocephalum* plants was far less than that of the monoterpenes and sesquiterpenes. However, it is noteworthy that komaroviquinone, isolated by Uchiyama et al. (2003) from *Dracocephalum komarovii* Lipsky in the early 21st century, has gained particular prominence. In the context of Chagas disease treatment, the natural product komaroviquinone and its derivatives have emerged as promising candidates, offering a novel therapeutic strategy. This study highlights the efficacy of komaroviquinone, which has shown significant anti-trypanosomal activity against Trypanosoma cruzi, the causative agent of Chagas. The potency of this metabolite is especially remarkable, as it demonstrates higher antiprotozoal activity than the standard drug benznidazole, without concurrent toxicity to host cells (Suto et al., 2015). *Dracocephalum forrestii* W.W.Sm. is currently the plant with the most abundant triterpene metabolites, whereas betulinic acid and ursolic acid have been detected in three or more *Dracocephalum* species. During plant desiccation, there may be a general accumulation phenomenon of these antioxidant-rich triterpenoid metabolites (Abbasi et al., 2019). This antioxidant effect originates from the plants themselves, indicating that throughout evolution, plants have developed the capability to synthesize these compounds to protect themselves from oxidative stress.

### 3.2 Flavonoids

In *Dracocephalum*, 160 flavonoid metabolites were collected, including 94 flavone, 44 flavonol and 22 dihydroflavonoid metabolites. The pioneering documentation of flavonoids within the *Dracocephalum*, as found in current literature, originates from the work of Shamyrina et al., who characterized the presence of apigenin and its glucoside derivative, apigenin 7-*β*-D-glucopyranoside from *D. nutans* (Shamyrina et al., 1975). Subsequently, metabolites such as cosmosiin, pedalitin, and pedaliin were successively isolated from *D. tanguticum* by Zhang et al. (1994). Flavonoids were most widely distributed in vanilla, including isorhamnetin, kaempferol, tilianin, xanthomicrol, 8-hydroxy-salvigenin, chrysoeriol, etc (Sharafi et al., 2014; Zhang et al., 2019). The most widely distributed metabolite are luteolin and its derivatives, which have been identified in *Dracocephalum* (Sharafi et al., 2014). Cosmosiin has also been identified in *D. moldavica*, *D. tanguticum*, and *D. palmatum* (Zhang et al., 2019). Most flavonoids are distributed throughout the plant, but some tend to accumulate in the tissue of specific species. For example, Apigenin-7-*O*-glucoside is predominantly accumulated in the leaves of *Dracocephalum rupestre* Hance (Ferreira et al., 2016); (*2S*)-Isosakuranetin 7-*O*-*β*-D-(6ʺ-o-malonyl) glucopyranoside and (*2S*) -Poncirin can only be detected in the aboveground part of *Dracocephalum fruticulosum* Stephan ex Willd. (Sabrin et al., 2021); chrysoeriol *O*-*β*-D-glucopyranoside was specifically distributed in the aboveground part of *D. nutans* (Conrad et al., 2009); acacetin-7-*O*-(3-*O*-malonyl)-*β*-D-glucopyranoside and acacetin-7-*O*-(2-*O*-malonyl)-*β*-D-glucuronopyranoside are distributed in the aboveground portion of *Dracocephalum foetidum* Bunge (Selenge et al., 2014). In addition, several flavanone glucosides deserve attention. For example, (*2S)* -isosakuranetin 7-*O*-*β*-D-(6ʺ-o-malonyl) glucopyranoside, (*2S*) -poncirin, and isosakuranin, present in *D. fruticulosum*, naringenin-7-*O*-*β*-D-glucuronopyranoside, present in *D. palmatum* (Sabrin et al., 2021; Olennikov et al., 2013). Naringenin-7-*O*-*β*-D-glucuronopyranoside is one of the main substances controlling the bitter taste of plants, which may be a factor affecting the flavor of medicinal materials (Matoušek et al., 2012).

### 3.3 Chemical metabolites of the first classification

Over the past 20 years, investigators of the *Dracocephalum* genus have made significant strides in successfully isolating and characterizing a plethora of new metabolites. In addition to the aforementioned komaroviquinone, Saeidnia et al. reported the isolation of two novel monoterpene glycosides from *D*. *kotschyi*: limonen-10-ol 10-*O*-*β*-D-glucopyranoside and limonen-10-ol 10-*O*-*β*-D-glucopyranosyl-(1→2)-*β*-D-glucopyranoside. Regrettably, these metabolites did not exhibit the potent trypanocidal activity observed for limonene-10-aL (Saeidnia et al., 2004). In 2008, Dai et al. extracted 1′-methyl-2′-hydroxyethyl ferulate (a ferulic acid ester) from *Dracocephalum peregrinum* L., which demonstrated a modest inhibitory effect on nitric oxide (NO) production. Cellular viability assays using MTT [3-(4,5-dimethylthiazol-2-yl)-2,5-diphenyltetrazolium bromide] indicated that this metabolite did not significantly affect RAW264.7, at efficacious concentrations (Dai et al., 2008).

In 2009, Fu et al. identified three flavonoid glycosides (Peregrinumin A, B, and C) and one cyanogenic glycoside (Peregrinumcin A) from *D*. *peregrinum*. These metabolites displayed commendable anti-inflammatory activity *in vitro*, effectively suppressing the production of nitric oxide (NO) in RAW 264.7 and 293 cells activated by lipopolysaccharide (LPS). Notably, Peregrinumin A and Peregrinumcin A exhibit robust anti-inflammatory effects at a dose of 100 mg/mL (Fu et al., 2009). In the same year, Wang et al. isolated four novel spermidine glycosides, dracotanosides A-D, from *D*. *tanguticum*, yet their biological activities and functions were not elucidated (Wang et al., 2009). In 2009, Li et al. discovered four new metabolites from *D*. *forrestii*, among which only 3,4,5-trimethoxyphenylethanol *β*-D-glucopyranoside demonstrated modest anti-inflammatory effects (Li et al., 2009).

In 2011, Zeng et al. reported that among the four glycosides found in *D*. *tanguticum*, benzyl-6-[(*2E*)-2-butenoate]-*β*-D-glucopyranoside exhibited moderate inhibitory activity against NO with an IC_50_ value of 64.33 µM (Zeng et al., 2011). In 2017, Deng et al. identified two metabolites from *Dracocephalum taliense* Forrest ex W.W.Sm., one being the abietane diterpenoid 12-methoxy-18-hydroxy-sugiol and the other a highly oxygenated ursane triterpenoid named 2*α*,3*α*-dihydroxy-11*α*,12*α*-epoxy-urs-28,13*β*-olide. While these did not show significant anti-inflammatory activity, the latter metabolite displayed potential antitumor activity, exhibiting marked cytotoxic effects on the HepG2 (human liver cancer cell line) and NCI-H1975 (human lung adenocarcinoma cell line) with IC_50_ values of 6.58 ± 0.14 µM and 7.17 ± 0.26 µM, respectively (Deng et al., 2017). In 2019, Ma et al. discovered three new phenylacetamide glycosides, dratanguticumides A-C, from *D*. *tanguticum*. These metabolites showed moderate antihyperglycemic activity, as determined by the glucose consumption rate in 3T3-L1 adipocytes, with rates of 20.80% ± 1.47%, 21.48% ± 2.44%, and 21.57% ± 1.35%, respectively (Ma et al., 2020). In 2021, Zhang et al. identified eight lignans from *D*. *moldavica* whose biological activities remain to be elucidated (Zhang et al., 2021).

## 4 Ethnopharmacological uses

The genus *Dracocephalum*, along with its closely related genera *Hyssopus* and *Lallemantia*, presents a rich tapestry of ethnomedical uses across various plant parts such as the roots, stems, leaves, flowers, and seeds. Our comprehensive compilation verified 32 medicinal species within *Dracocephalum*, complemented by four within *Hyssopus* and two within *Lallemantia*, all taxonomically confirmed in the field of botanical science. The application of the whole herb is notably dominant in *Dracocephalum* and *Hyssopus*, representing a substantial 71.9% and 75.0% of the medicinal species from these genera, respectively. Conversely, the medicinal accounts for *Lallemantia* are solely attributed to its seeds (Table 2). To delineate the traditional medicinal attributes of these genera in more detail, we draw on the previous classification (Li et al., 2012a). The traditional medicinal properties have been categorized into 11 therapeutic applications, which encompass a spectrum of conditions, including gastrointestinal diseases; musculoskeletal issues; cardiovascular diseases; diabetes and urological disorders; gynaecological conditions; ailments of the ears, throat, mouth, and eyes; dermatological conditions; liver and gall bladder diseases; respiratory diseases; colds and fevers; and others (see Supplementary Table S2). We have consolidated this information and presented it visually in Figure 3, which clearly demonstrates that the traditional efficacy of these three genera is predominantly concentrated in addressing respiratory diseases, liver and gall bladder diseases.

### 4.1 Tibetan plateau and adjacent regions ethnomedicines

Some *Dracocephalum* species are distributed across the QTP and its surrounding regions, establishing themselves as an indispensable element of local and regional ethnopharmacopeia. These plants have become an integral part of traditional medicine for neighboring ethnic groups and countries, underscoring their importance in the ethnomedical landscape.

In Tibetan pharmacopeias dating back to the 17th century, a record exists of a Tibetan medicinal material known as “Priyangu,” characterized by its flowers that resemble fluttering blue flags and its whole plant possessing effects in alleviating liver heat and hemostasis. In contemporary Tibetan medicine, *D*. *tanguticum* isconsidered the mainstream variety of “Priyangu” (Tashi et al., 2022). However, some scholars from China’s ethnic minorities suggest that because of ambiguous botanical descriptions in ancient Tibetan medical literature, *Dracocephalum calophyllum* Hand.-Mazz. and *Dracocephalum isabellae* Forrest ex W.W.Sm. may also be used as “Priyangu” to alleviate liver heat (He et al., 2015). Unfortunately, literature regarding the chemical metabolites and pharmacological properties *D. calophyllum* and *D. isabellae* is lacking.

*D*. *forrestii* is a Tibetan medicinal plant that is indigenous to the mountainous regions of Yunnan Province, China. The aerial parts of *D. forrestii* are used as diuretic, astringent, and antipyretic agents (Weremczuk-Jeżyna et al., 2020). Given its use as a substitute for *D. tanguticum*, *D. forrestii* has emerged as a medicinal plant that warrants in-depth investigation of its pharmacological activities and clinical applications. *D. taliense* is a species endemic to Yunnan Province. Local communities utilize the entire plant of *D. taliense* for the treatment of hepatic disorders. The plant has demonstrated significant efficacy particularly in cases of hepatitis and jaundice, as well as for the regulation of gastrointestinal health (Deng et al., 2017).

*D*. *heterophyllum* is recognized as one of the traditional Tibetan medicinal plants within the *Dracocephalum* genus. In the traditional Tibetan medical system, *D. heterophyllum* is also known as “Ao-Ga” or “Ji-Mei-Qing-Bao,” and has been utilized by Tibetan physicians to treat various conditions, including jaundice, liver diseases, coughs, lymphangitis, oral ulcers, and dental disorders (Li et al., 2022). Additionally, the Uyghur people employ *D. heterophyllum* to treat certain cardiovascular and respiratory diseases (Jiang et al., 2018).

*D. integrifolium* is predominantly found in Central Asia and is referred to as “Marzan Juxi” in traditional Uyghur herbal medicine, where it is used for the treatment of coughs and asthma (Zhou et al., 2019). *D. peregrinum* is widely distributed across Russia, Mongolia, and Northern China. In the traditional medicine of the Kazakh people in Xinjiang, *D. peregrinum* is known as “Tekanbasjelanbas,” and the entire plant is commonly used for treating colds and liver diseases (Yan et al., 2020; Dai et al., 2008).

### 4.2 Ethnopharmacological heritage beyond the Tibetan plateau

Beyond the QTP and its surrounding regions, we provide a comprehensive overview of the utilization of *Dracocephalum* medicinal plants in other countries and regions.

For example, *D. rupestre* is widely distributed across various provinces in China, including Hebei, Shanxi, Qinghai, Inner Mongolia, and Liaoning. According to records in the “Chinese Herbal Medicine Dictionary,” the entire plant of *D. rupestre* is used to treat conditions such as externally contracted wind-heat, headache with chills fever, cough, jaundice, and hepatitis. Additionally, the tender stems and leaves of *D. rupestre* form a traditional Chinese medicinal food, known as “Maojian” tea (Wang et al., 2022).

*D. austriacum*, and *D. botryoides* are characteristic *Dracocephalum* species found in the Caucasus region. In Azerbaijan, these two species are considered medicinal. Local populations add the aerial parts of these plants into noodle soups to treat respiratory and gastrointestinal disorders. In local folk medicine, herbal decoctions of *D. botryoides* are used to treat liver diseases, gastritis, and ulcers, whereas those of *D.* austriacum possess anti-inflammatory properties and aid in wound healing (Kashchenko et al., 2022).

*D*. *komarovii* predominantly grows in the high-altitude regions of the Western Tian Shan Mountain range. In Uzbekistan, *D*. *komarovii* is known as “buzbosh,” and the local population utilizes the aerial parts of the plant to prepare a tea that is traditionally consumed to address various ailments, including inflammatory conditions and hypertension (Uchiyama et al., 2006).

*D*. *kotschyi* is a traditionally used medicinal plant in Iran, referred to as “Zarrin-giah” in Persian. In the traditional Iranian medical system, this plant is used for its antispasmodic, antihyperlipidemic, and analgesic properties. It is also used to treat symptoms such as headaches, fever, inflammation, bruising, rheumatism, gastric disorders, and liver diseases, and serves as an analgesic for complications related to kidney function. In Iran, it is boiled to alleviate rheumatic pain and to facilitate wound healing. Furthermore, it is recognized for its capacity to strengthen the immune system (Ghavam et al., 2021; Heydari et al., 2019).

From the Caucasus to Siberia and China, the whole herb or seedling of *D. calophyllum* has been reported to have effects on clearing the heat of the liver, stomach, and lungs, stopping bleeding, healing sores, eliminating dampness, relieving itching, and treating dizziness, visceral pus, prurigo rheumatism, hematochezia, hematuria, sore mouth incompatibility, edema, and ascites (Jiang, 2015).

*D. moldavica* is recognized as a *Dracocephalum* species with the most extensive folk medicinal applications, for which we collected data. In the northern Iranian Boulze Mountains, *D. moldavica* is commonly known by the name ‘Badarshoo.’ It is used as a food additive in yogurt or as a processed herb. It can effectively treat stomach and liver diseases, headaches, and congestion. In regions spanning Eastern Europe, Siberia, Mongolia, and China, *D*. *moldavica* has been used extensively for its traditional therapeutic properties. These include its applications in clearing heat and detoxifying, cooling the blood and purging internal ‘fire,’ alleviating pain, arresting bleeding, and providing relief from cough and asthma. Additionally, it is recognized for its expectorant effects and capacity to protect liver health (Morshedloo et al., 2020; Zhang et al., 2018). In China, *D. moldavica* is the main composition in certain clinical preparations, such as Qinggan Qiwei powder, Niuhuang Shisanwei pill, and Liganhewei pill (Xing et al., 2021).

In addition, *H. officinalis* essential oils have therapeutic effects on coughing, loss of appetite, fungal infections, spasmodic diseases, and antibacterial activity. The biological activity and aroma of essential oils indicate that they can serve as potential antioxidant food ingredients (Sharifi-Rad et al., 2022). Various compositional metabolites of *L. iberica* and *L. royleana* seeds such as proteins, oils, fatty acids, and carbohydrates play a significant role in their commercial value of the seeds. The oils, composition of fatty acids (linolenic acid, linoleic acid, and oleic acid), and mucilage are the main resources in *Lallemantia* seeds that are used by the food and pharmaceutical industries (Paravar et al., 2023)*. L. royleana* is used as a diuretic, nourishing, aphrodisiac, and cough suppressant in traditional and folk medicine in Iran, and is used to treat various neurological, liver, and kidney diseases (Mohammed et al., 2022). The traditional application of the plant indicates that the medicinal value of *Dracocephalum* mainly focuses on clearing away heat and protecting the liver, which echoes the anti-inflammatory and hepatoprotective effects of *Dracocephalum* in modern pharmacological research, possibly through phenols, flavonoids, and terpenoids in *Dracocephalum*. Therefore, combining the anti-inflammatory and hepatoprotective effects of *Dracocephalum* in clinical practice, and effectively improving its clinical utilization rate will be the focus of *Dracocephalum* plant research.

## 5 Pharmacological activities

The pharmacological properties of *Dracocephalum* have attracted considerable attention in recent years. The main pharmacological activities of *Dracocephalum* include hepatoprotective, anti-inflammatory, antimicrobial, anti-hyperlipidemic, and antitumor. In addition, recent research has reported novel pharmacological effects. Table 3 lists some *in vitro* and *in vivo* pharmacological models and related dosage information used to clarify the pharmacological activities of *Dracocephalum*. The main pharmacological activities of *Dracocephalum* are as follows.

### 5.1 Hepatoprotective

Elevated levels of serum alanine aminotransferase (ALT) and aspartate aminotransferase (AST) are important markers of LPS and CCl4-induced liver injury (Weber et al., 2003; Xiong et al., 2017). Zhu et al. (2018) established a Kunming mice liver injury model induced by acute CCl_4_ and confirmed that the extract of phenols from *D. rupestre* reversed the increase in serum ALT and AST levels induced by CCl_4_ in a dose-dependent manner, and lowered them to near-normal levels (Zhu et al., 2018). *D. heterophyllum* can reduce plasma ALT, AST, IFN-γ, and TNF-α concentration in experimental autoimmune uveitis (EAU) mouse models at a dose of 20 mg/kg, indicating that *D. heterophyllum* has certain advantages in protecting against liver damage (Zheng et al., 2016). The MTT cell viability experiment showed that the extract of *H. officinalis*, at a concentration of 500 μg/mL, did not exhibit hepatotoxicity in mouse FL83B hepatocytes. Furthermore, this concentration of the *H. officinalis* extract displayed a protective effect against both palmitic acid-induced and acetaminophen-induced hepatotoxicity. This suggests that it may contribute to the mitigation or prevention of hepatocellular damage induced by these chemical agents to a certain extent.

### 5.2 Anti-inflammatory activity

Anti-inflammatory drugs are the second largest class of drugs after anti-infective drugs. Therefore, exploring the anti-inflammatory effects of natural products has become a new trend in the research and development of novel anti-inflammatory drugs (Li et al., 2012b). The anti-inflammatory effects of *Dracocephalum* have also been extensively studied, including the anti-inflammatory mechanism underlying its traditional use for treating typhoid fever, common cold, cardiovascular diseases, gastritis, sore throat, rheumatism, pulse disease, and scabies (Sadraei et al., 2017; Nie et al., 2021).

Current research has shown that at the third and fourth hours after administration of carrageenan, the highest dose of 200 mg/kg of methanol extract from *H. officinalis* has a significant inhibitory effect on rat foot edema (Mićović et al., 2022). *In vitro* inflammatory models are often established using LPS-stimulated macrophages (RAW 264.7 cells). Toshmatov et al. isolated new Monoterpene glucosides—komarovin B and komarovin C—from *D. komarovii*, and found that they inhibited LPS-induced NO production in macrophages to alleviate inflammation at concentrations of 1, 10, 50, and 100 μM (Toshmatov et al., 2019). The anti-inflammatory experiment using RAW 264.7 cells showed that dracocephalumoid A, uncinatone, trichotomone F, and caryopterisoid C from *D. moldavica* can inhibit LPS-induced TNF-α, IL-1β or the production of NO has a significant inhibitory effect, with IC_50_ values ranging from 1.12 to 5.84 μM (Nie et al., 2021).

### 5.3 Antimicrobial

Studies have shown that *D. moldavica* contains geranyl acetate, geranial, and neral, and can effectively inhibit *Staphylococcus aureus*, *Escherichia coli*, *Salmonella typhimurium*, and *Listeria monocytogenes* (Aćimović et al., 2022). Essential oil of *H. officinalis* effectively shows antifungal activity at concentrations of 500 and 1,000 μg/mL, with the main active metabolites being isopiperitenone, pinocampheol, and *α*-pinene (Harčárová et al., 2021). Rosmarinic acid, methyl rosmarinate, butyl rosmarinate, and salvigenin significantly inhibit *E*. *coli*, *Candida albicans*, and *S*. *aureus* growth in *H. cuspidatus* (Shomirzoeva et al., 2020). The antibacterial activity of *D. kotschyi* is mainly indicated by its inhibition of *S. aureus* and *E. coli* (Moridi Farimani et al., 2017). *L. iberica* showed antibacterial activity against *E*. *coli*, *Pseudomonas aeruginosa*, *S*. *aureus* and *Enterobacter aerogenes*, and the largest inhibitory activity was observed against *P. aeruginosa* and *E. aerogenes* (Yilmaz Kardas et al., 2023).

### 5.4 Antihyperlipidemic

Through a rat model, Aslian et al. validated that the *D. kotschyi* can upregulate the expression of p-FOXO1, p-AKT, and PPARγ and downregulate the expression of FOXO1, p-JNK, and SREBP-1 at concentrations of 0.25 and 0.5 mL/rat to exert antihyperlipidemic activity. In the pathogenesis and treatment of hyperlipidemia, multiple key signaling factors play significant roles. PPARγ, as a primary regulator of adipocyte differentiation and lipid metabolism, can promote the storage of fatty acids and the breakdown of lipids when activated, contributing to the reduction of blood lipid levels. FOXO1, acting as a transcription factor, affects lipid metabolism by inhibiting the activity of PPARγ. Phosphorylated FOXO1 (p-FOXO1) is usually associated with the suppression of FOXO1 activity, and its increase may help to reduce the negative impact of FOXO1 on lipid metabolism. Additionally, SREBP-1 influences blood lipid levels by regulating the synthesis of cholesterol and fatty acids. The activation of the AKT signaling pathway, particularly in the form of p-AKT, is crucial for improving insulin resistance and regulating lipid metabolism. The activation of p-JNK is often related to cellular stress responses and inflammatory reactions, and it may exacerbate insulin resistance by promoting the activation of FOXO1 (Aslian and Yazdanparast, 2018). The total flavonoid extract of *D. moldavica* can significantly improve hyperlipidemia in rats by regulating TG, LDLC, HDLC, ICAM-1, VCAM-1, PCNA, and other indicators at doses of 21, 42, and 84 mg/kg and exhibited a dose-dependent effect (Quan et al., 2017). In addition, the study on the protective effects of *L. royleana* seed polysaccharides against liver and kidney injury in rats induced by a high-cholesterol diet indicates their potential to safeguard hepatic and renal functions and tissue integrity, thereby providing a novel avenue for natural products in the management of hyperlipidemia (Mohammed et al., 2022).

### 5.5 Antitumor

Generally, when evaluating the antitumor activity of natural products, we mainly investigate their ability to inhibit tumor cell proliferation and promote immune cells to secrete cytokines acting on tumor cells (Zhang et al., 2019). Studies have shown that the methanolic extract of *H. officinalis* can increase apoptosis, reduce cell division, reduce tumor volume, and prolong survival in C6 glioma cells, mainly due to the upregulation of p53 and p21 mRNA expression (Khaksar et al., 2022). The *D. taliense* was tested for its antitumor activity against HepG2 cells using flavonoids at concentrations of 10, 20, 40, and 80 µM (Deng et al., 2017).

### 5.6 Other pharmacological activities

The hypoglycemic effects of *D. tanguticum* were confirmed in 3T3-L1 cells. It exerted moderate effects at a final concentration of 25 μM (Ma et al., 2020). The total flavonoids of *D. moldavica* (25, 50, 100 mg/kg/d) can upregulate MDA, SOD, and GSH-Px expression and downregulate IL-6, IL-8, and TNF-α expression in the brain tissue of model rats, effectively improving myocardial ischemia (Su et al., 2010). Frosmaric acid and oleanolic acid in *D. moldavica* also improve memory impairment (Deepa et al., 2020), whereas tilianin, luteolin, and apigenin improve vascular dementia (Liu M. et al., 2021). The antiviral effects of isosakuranetin glycosides and phenylpropanoid oligomers from *D. foetidum, D. nutans,* and *D. fruticulosum* have been confirmed through *in vitro* experiments (Sabrin et al., 2021). In animal models of systemic and local allergic reactions, Kim et al. found that the water extract of *D*. *argunense* regulates TNF-α and IL-6 in a dose-dependent manner, indicating its anti-allergic effect (Kim and Shin, 2006).

## 6 Discussion

**FIGURE 5:**
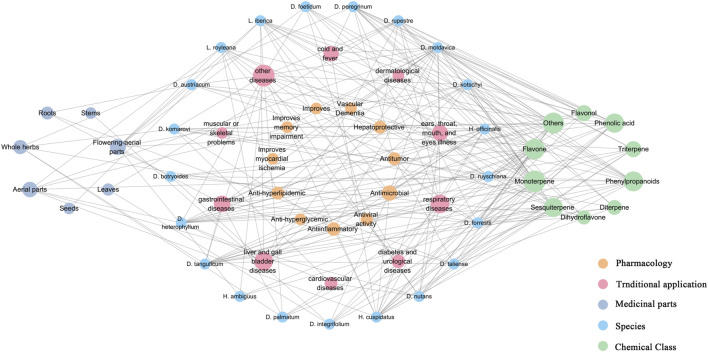
Network analysis of genus *Dracocephalum*-chemical metabolites-traditional application and pharmacological activities. (We used Microsoft Excel 2010 software for statistical analysis and Cytoscape 3.7.2 software for visual presentation and established a network diagram. Nodes represent medicinal plants, chemical metabolites, traditional applications, and medicinal parts of *Dracocephalum*, and edges are connected to represent their mutual relationship; Connectivity indicates the number of edges directly associated with a node in the network diagram. The greater the connectivity, the wider the network of the node.

*Dracocephalum* is a large and complex group of plants, and the intricate relationships between it and closely related genera can be discerned from the classification methods applied by scholars throughout history (Chen Y. P. et al., 2022). Preliminary pharmacophylogeny studies suggest that a certain regularity exists among medicinal plants of the genus *Dracocephalum*; species that are phylogenetically closer tend to exhibit some aggregation in terms of geographical distribution, chemical metabolites (the distribution pattern of these metabolites), and therapeutic effects (as reflected in ethnopharmacology and pharmacological activity) (Abbasi et al., 2022). We used Microsoft Excel 2010 software for statistical analysis and Cytoscape 3.7.2 software for visual presentation, establishing a network diagram (Figure 5). This diagram integrated data on plant species, metabolite profiles, pharmacological activities, traditional uses, and medicinal components of 21 medicinal plants from genus *Dracocephalum* and its related genera for a visual analysis. This approach facilitated the exploration of their untapped potential. *D. moldavica*, *D. heterophyllum*, *D. forrestii*, *H. cuspidatus*, and *H. officinalis* all show higher connectivity. Extensive research has been conducted on the metabolites and pharmacological effects of these species, which have significant traditional applications and are known for their terpenoids, flavonoids, and phenylpropanoids with high chemical connectivity. These metabolites have been found to possess hepatoprotective properties, as well as anti-inflammatory, antitumor, and antimicrobial effects (Figure 5). *D. moldavica* and *H. cuspidatus* had the most reported types of metabolites, with relatively balanced proportions. *D. heterophyllum*, *D. kotschyi*, and *D. forrestii* have several reported metabolites, particularly terpenoids in the studied types. In contrast, reports on *D. austriacum*, *D. rupestre*, *D. botryoides*, and *D. palmatum* are predominantly related to their flavonoid and phenylpropanoid contents. This phenomenon indirectly reflects the medicinal tendencies of these species, illustrating a potential dialectical relationship between the traditional efficacy and metabolite diversity.

The phylogenetic tree established by Chen G. W. et al. (2022) featured nine clades, eight of which contained species of medicinal varieties. However, the functions of many of these medicinal plants are only documented in traditional applications, without clear pharmacological mechanisms. *D. moldavica* is recognized as the *Dracocephalum* species with the richest known diversity of compounds and the most extensive pharmacological activities. However, some of its traditional medicinal claims remain unverified. Phylogenetic analysis has demonstrated that *D. moldavica*, *D. heterophyllum*, and *D. kotschyi* are closely related. In traditional ethnomedical practices, these species are utilized for the treatment of liver and gall bladder diseases. Specifically, *D. moldavica*, *D. heterophyllum*, and *D. kotschyi* are reputed to possess therapeutic effects against hepatitis, jaundice, and liver pain, respectively. However, in our survey, we identified only studies concerning the ethanol extract of *D. heterophyllum* and its impact on ConA-Induced Acute Hepatitis. Therefore, many extended discussions on the efficacy of medicinal plants are based on traditional applications. Medicinally related species include *D. foetidum* and *D. komarovii*, as well as some closely related plants that have not yet been documented, including *Dracocephalum stamineum* Kar. & Kir., *Dracocephalum renati* Emb., and *Dracocephalum spinulosum* Popov. The lack of documentation for these species may be attributed to their perceived lack of significant medicinal properties or because their potential remains undeveloped or has not been fully investigated. Medicinal plants, such as *D. nutans*, *D. integrifolium*, and *H. cuspidatus* are closely related and form a single branch in the phylogenetic tree. They are all effective in treating respiratory diseases; therefore, we speculate that closely related species such as *Dracocephalum psammophilum* C.Y.Wu & W.T.Wang and *Hyssopus seravschanicus* (Dubj.) Pazij may have a similar efficacy. Similarly, the clade comprising *D. calophyllum*, *D. forrestii*, *D. taliense*, and *D. tanguticum* is known to exhibit therapeutic effects on gastrointestinal conditions, raising the question of whether *Dracocephalum propinquum* W.W.Sm. and *Dracocephalum microphyton* Y.P.Chen, Y.S.Chen & C.L.Xiang share similar beneficial properties, meriting further investigation. Furthermore, although neurological disorders are grouped within the category of “Other diseases,” the shared efficacy of *L. royleana* and *L. iberica* in addressing neurological as well as hepatic and renal diseases is particularly noteworthy. Consequently, the closely related species *Lallemantia peltata* (L.) Fisch. & C.A.Mey. and *Dracocephalum scrobiculatum* Regel should also garner the interest of researchers because of their potential applications in this area. In addition, *D. rupestre*, which has the potential for use in medicine and food, can be used as a tea. According to the theory of consanguinity, we speculate that *D. bipinnatum* and *Dracocephalum adylovii* I.I.Malzev are likely homologous plants used in medicine and food. Therefore, there is an urgent need to provide a suitable theoretical and technical system for medicinal and edible homologous items, enhance the scientific connotations of medicinal and edible homologous items, and allow people to use medicinal and edible homologous items to meet the needs of contemporary health and disease (Hao et al., 2022; He et al., 2022; Zhang et al., 2022; Pan et al., 2022; Selvaraj and Gurumurthy, 2022; Yao et al., 2022).

The genus *Dracocephalum* originated from the steppe-desert biomes of Central and West Asia, and the alpine region of the QTP. It has a long history of medicinal uses. Previous studies showed that this genus spread from East and West Asia to QTP alpine region during the Pliocene. Drought on the Eurasian continent has promoted rapid radiation in the region, and the uplift of QTP has promoted the distribution and diversity of species in this genus. *D. forrestii* occurs only in the northwestern mountains of Yunnan Province, China. *D. taliense* is distributed across Dali, Yunnan. *D. integrifolium* is found primarily in Xinjiang, China. *D. nutans* is found throughout Siberia, Eastern Europe, and Kashmir. It is also seen in Heilongjiang, Inner Mongolia, and northern Xinjiang. *D. moldavica*, and *D. heterophyllum* are found in Inner Mongolia, Gansu, Qinghai, and in Eastern Europe. *Dracocephalum paulsenii* Briq., and *Dracocephalum origanoides* Stephan ex Willd. are distributed in Xinjiang and Russia. *Hyssopus latilabiatus* C.Y.Wu & H.W.Li, *H. cuspidatus*, *D. integrifolium*, and *D. nutans* are distributed in Xinjiang. *H. officinalis* is distributed in Europe, Algeria, Morocco, Iran and other places, whereas *D. moldavica*, *D. rupestre*, *D. heterophyllum*, and *H. officinalis* overlap in Inner Mongolia, Shanxi, Gansu, and Qinghai. *H. cuspidatus* has been found in North Africa, India, Mongolia, Russia, and China (Deepa et al., 2020). *L. iberica* originated in the Caucasus and the Middle East and is widely distributed in some western parts of Europe and Asia, while *L. royleana* grows in Iran and almost all parts of Middle East. The genus *Hyssopus* is widely distributed in Central and West Asia, Siberia, and Europe, whereas the genus *Lallemantia* is distributed in West Asia and *L. royleana* is further extended to Central Asia, which is consistent with our research. We preliminary verified the rationality of combining *Hyssopus* and *Lallemantia* with *Dracocephalum* through the spatial distribution pattern map and the suitable Ecotope (Figure 1). From the perspective of spatial distribution patterns, the distribution of genus *Dracoephalum* is wider than that of *Hyssopus* and *Lallemantia*, but the two have overlapping distributions in the European region. From the perspective of suitable Ecotope, *Dracocephalum*, *Lallemantia* and *Hyssopus* were suitable for growing in western North America and western Asia. Consistent with the results of our previous phylogenetic studies, *Sect. Dracocephalum*, *Sect. Sinodracon*, and *Sect. Keimodracon* are phylogenetically close to each other and can be used to verify the phylogenetic relationships between the *Hyssopus*, *Lallemantia*, and *Dracocephalum* genera at the geographical distribution level (Chen Y. P. et al., 2022).

*Dracocephalum grandiflorum* L., *D. integrifolium*, *D. nutans*, *D. origanoides*, *D. paulsenii*, *D. forrestii*, *D. heterophyllum*, *D. moldavica*, *D. grandiflorum*, *D. bipinnatum*, *D. peregrinum*, *D. rupestre*, *Dracocephalum rigidulum* Hand.-Mazz., *L. iberica* and *Hyssopus* species share similarities in clearing heat, detoxifying, cooling blood, relieving bleeding, treating bronchitis, and relieving cough and asthma. *D. forrestii* has traditional efficacy in the treatment of cardiovascular diseases and rheumatoid arthritis, which has not yet been proven in pharmacological studies. Both modern pharmacological research and traditional application have shown that *D. rupestre*, *D. nutans*, *D. taliense*, *D. tanguticum*, *D. peregrinum*, *D. heterophyllum*, *H. officinalis*, *L. iberica*, and *L. royleana* possess hepatoprotective activities. The Encyclopedia of Chinese Medicine records that the aboveground parts of *D. moldavica* are used to treat all liver disorders (Editorial Board of Chinese Medical Encyclopedia, 1992). Terpenoids and flavonoids are abundant in closely related species, among which, monoterpenes are the most common, and the dominant flavonoids are flavones and their glycosides (Table 1). We speculate that the traditional therapeutic properties of clearing heat and detoxifying, treating bronchitis, relieving cough, and relieving asthma may be related to the content of monoterpenes and flavonoids in *D. heterophyllum*, *D. rupestre*, *D. forrestii*, *D. integrifolium*, *D. nutans*, *D. origanoides*, *L. iberica*, *H. latilabiatus*, *H. cuspidatus*, which supported the anti-inflammatory activity of *H. cuspidatus* to a certain extent. According to the theory of pharmacosystematics, *D. integrifolium*, *D. nutans*, *D. rigidulum*, *D. origanoides*, *D. forrestii*, and *L. royleana* may be a new potential resource for the development of anti-inflammatory drugs. Both *L. royleana* and *D. moldavica* exhibit antibacterial, and anti-hyperlipidemic activities, possibly owing to the presence of soluble fiber and polysaccharides. Moreover, triterpenoids were concentrated in *H. cuspidatus*, *D. taliense* and *D. forrestii* of *Sect. Sinodracon*, but *Ser. Angustifolii* and *Sect. Sinodracon* and *Sect. Idiodracon* exhibited antibacterial, anti-tussive, and anti-asthmatic effects, implying that known triterpenoids might not be the only active substances, and other effective metabolites are yet to be identified (Tables 2, 3). Both *Sect. Calodracon* and *Sect. Dracocephalum* contains essential oils, seven-substituted flavonoid glycosides and three-substituted flavanol glycosides (Table 1). *Ser. Officinales* Boris., *Sect. Calodracon*, and *Sect. Dracocephalum* have the common effects of protecting the liver, clearing heat and toxic materials, relieving cough and asthma, and eliminating phlegm. Additionally, *Sect. Calodracon* and *Ser. Officinales* Boris. have the common effects of cooling the blood, and purging fire. Based on these effects, *Sect. Calodracons* can also prevent bleeding and promote granulation. Therefore, these phylogenetically close species may have similar pharmacodynamic bases, such as chemical composition and therapeutic effects, which are relevant to their clinical efficacy. Phytochemistry research has identified 19 species of *Dracocephalum*, *Lallemantia*, and *Hyssopus*, and found that most of them contain terpenoids, flavonoids, and phenylpropanoids. These metabolites are key substances for medicinal plants to exert pharmacological effects, with good pharmacological activity and potential anti-inflammatory, and hepatoprotective effects. In addition, plant metabolites not only have pharmacological effects but also determine their traditional applications. The genus *Dracocephalum* is widely used for the treatment of cough, fever, and liver and gall bladder diseases. Owing to the similar pharmacological activities of the three genera, *Lallemantia* and *Hyssopus* have similar traditional therapeutic effects. Based on the latest phylogenetic and biogeography studies of *Dracocephalum*, we provide further evidence for the merger of *Lallemani*a and *Hyssopus* into *Dracocephalum* and lay a foundation for finding plants with the medicinal value of this kind in the future.

**TABLE 1:** Distribution of specialized metabolites in *Dracocephalum, Hyssopus*, and *Lallemantia.*

Section/Series	Species	Terpenoids				Flavonoid			Phenylpropanoids	Phenolic acid	Others
		Monoterpene	Sesquiterpene	Diterpene	Triterpene	Flavone	Flavonol	Dihydroflavone			
*Sect. Dracocephalum*	*D. moldavica*	※**	※	***	*	****	**	*	※****	****	※**
	*D. heterophyllum*	※※***	※※		**	**	*		**	*	※※**
	*D.peregrinum*	※	***		**	※	***	*	***	**	※
*Sect. Sinodracon*	*D. forrestii*	**	***	*	****	*		*	****	**	**
	*D. taliense*			***	*					*	*
	*D. tanguticum*	※*	****	*		***		*	***		※*
*Sect. Calodracon*	*D. rupestre*				*	**	*	**		*	*
*Sect.Idiodracon*	*D.integrifolium*	****	***								*
	*D. nutans*	※*	*								
*Sect. Ruyschiana*	*D.ruyschiana*					**	*		**	*	*
*Ser. Angustifoli*	*H. cuspidatus*	※***	*		*	***	***		※※	***	※**
*Ser. Officinales*	*H. officinalis*	※※**	****			**	*		*		*
Others	*D. palmatum*				*	***		**	**		
	*D. foetidum*	※*	*			***			**		
	*D. kotschyi*	※※****	※	*	*	**	*		*		***
	*D. austriacum*					***			****	***	
	*D. botryoides*					***		**	***	****	
	*D. komarovi*	*		*	*					*	*
	*L. iberica*	**	*						*	*	**
	*L. royleana*	※**	***								**

Note: *, 1–5 metabolites; **, 6–10 metabolites; ***, 11–15 metabolites; ****, 16–20 metabolites; ※, 21–25 metabolites; ※*, 26–30 metabolites; ※**, 31–35 metabolites; ※***, 36–40 metabolites; ※****, 41–45 metabolites; ※※, 46–50 metabolites; ※※*, 51–55 metabolites; ※※**, 56–60 metabolites; ※※***, 61–65 metabolites; ※※****, 66–70 metabolites.

**TABLE 2:** Geographical distribution and traditional applications of *Dracocephalum*.

Medicinal plant	Region (From the world plants)	Medicinal parts	Traditional therapeutic properties	References
*D. argunense*	Siberia (Chita); Russian Far East (Amur, Khabarovsk, Primorye); China (Hebei, Heilongjiang, Jilin, Liaoning, Nei Mongol); Mongolia; North Korea South Korea; Japan (Hokkaido, Honshu)	Aerial part, Whole herb	Gastritis, hepatitis, pulmonary phthisis	Saren and Saren (2002) Kim and Shin (2006)
*D. austriacum*	Switzerland; Austria; Czech Republic; Slovakia; Hungary; NE-Spain; France; Italy; Romania; C-European Russia; SW-Ukraine; Northern Caucasus; Georgia [Caucasus]; Armenia; Turkey (E-Anatolia, NE-Anatolia)	Whole herb, root	Stop bleeding, granulation promoting (wound healingremedy), decrease inflammation	Sokolov (1991) Kashchenko et al. (2022)
*D. bipinnatum*	Kazakhstan; Kyrgyzstan; Tajikistan; Afghanistan (Badakshan); China (Xinjiang); Tibet; Pakistan (Chitral, Astor, Gilgit, Baltistan, Hunza); NW-India (Jammu and Kashmir)	Stems, leaves, flowers	Relieve cough and asthma, clear heat	Jiang (2015)
*D. botryoides*	Armenia; Azerbaijan	Whole herb, root	Liver diseases, gastritis, and ulcers	Sokolov (1991) Kashchenko et al. (2022)
*Dracocephalum bullatum* Forrest ex Diels	China (Yunnan: Lijiang)	Whole herb	Secure the foetus	Jiang (2015)
*D. calophyllum*	China (Sichuan: Daocheng, Yunnan: Lijiang)	Whole herb, seedling	Liver heat; stomach heat; lung heat; prurigo rheumatism; stop bleeding, heal sore, eliminate dampness and relieve itching, dizziness, visceral pus, hematochezia, hematuria, edema and ascites	Jiang (2015)
*Dracocephalum discolor* Bunge	Siberia (Altai, Krasnoyarsk, Tuva); Kazakhstan; Kyrgyzstan; Mongolia	Stems, leaves, flowers	Relieve cough and asthma, clear heat	Jiang (2015)
*Dracocephalum diversifolium* Rupr	Kazakhstan; Uzbekistan; Kyrgyzstan; Tajikistan	Stems, leaves, flowers	Relieve cough and asthma, clear heat	Jiang (2015)
*D. foetidum*	Siberia (Altai, Krasnoyarsk, Tuva); Mongolia	Leaves, flowers	Fever; oral cavity diseases; rheumatic edema, and wounds, suppurative diseases, prevent bacterial and fungal infections	Lee et al. (2007) Selenge et al. (2014)
*D. forrestii*	China (Yunnan: Lijiang)	Whole herb, seedling	Liver heat; stomach heat; lung heat; prurigo rheumatism; stop bleeding, heal sore, eliminate dampness and relieve itching, dizziness, visceral pus, hematochezia, hematuria, edema and ascites	Jiang (2015)
*D. fruticulosum*	Siberia (Buryatia, Tuva); China (Ningxia); Mongolia	Aerial part	Sore throat; jaundice, liver heat, hepatitis; cold; cough; stomach heat, stomach spasm, stomach bleeding, dysentery; food poisoning, green leg disease, clearing heat and drying dampness, cool blood and stopping bleeding, wound healing, headache	Zhu (1993)
*D. grandiflorum*	Siberia (Altai, Buryatia, Chita, Irkutsk, Krasnoyarsk, Tuva, W-Siberia); Kazakhstan; Kyrgyzstan; Tajikistan; China (Nei Mongol, Xinjiang); Mongolia	Whole herb	Expelling phlegm and relieving asthma; clearing heat and detoxification	Jiang (2015)
*D. heterophyllum*	Kazakhstan; Kyrgyzstan; Tajikistan; Afghanistan (Wakhan); China (Gansu, Nei Mongol, Ningxia, Qinghai, Shanxi, Sichuan, Xinjiang); Tibet; Mongolia; NE-Pakistan (Deosai); Nepal; N-India (Himachal Pradesh, Jammu and Kashmir, Uttarakhand, Rupshu, Ladakh, Sikkim, Chumb)	Whole herb, stem, leaves, flowers	Cough, chronic bronchitis; goiter and tumor, mouth ulcers, web-eye; liver heat, jaundice; hypertension; clear heat and headache	Jiang (2015)
*D. imberbe*	Siberia (Altai, Krasnoyarsk, Tuva); Kazakhstan; Turkmenistan; Uzbekistan; Kyrgyzstan; Tajikistan; China (Xinjiang); Mongolia	Whole herb	Expelling phlegm and relieving asthma; clearing heat and detoxification	Jiang (2015)
*D. integrifolium*	Siberia (Altai); Kazakhstan; Uzbekistan; Kyrgyzstan; Tajikistan; China (Xinjiang); Mongolia	Whole herb, aerial parts	Expectorant, antitussive, antiasthmatic, senile chronic bronchitis	Jiang (2015)
*D. isabellae*	China (Yunnan: Zhongdian Shan)	Whole herb, seedling	Liver heat; stomach heat; lung heat; prurigo rheumatism; stop bleeding, heal sore, eliminate dampness and relieve itching, dizziness, visceral pus, hematochezia, hematuria, edema and ascites	Jiang (2015)
*D. komarovii*	Kazakhstan; Uzbekistan; Kyrgyzstan; Tajikistan	Aerial parts	Inflammatory diseases and hypertony	Uchiyama et al. (2003)
*D. kotschyi*	Iran (EC-Iran, N-Iran, S-Iran, W-Iran)	Whole herb, leaves	Stomachache; liver pain; various cancers, headache, relieve pain	Jiang (2015) Poursalavati et al., 2021 Khamesipour et al. (2021) Cham et al. (2022)
*D. moldavica*	Siberia (Chita, Krasnoyarsk, Tuva, W-Siberia); Russian Far East (Amur, Primorye); Turkmenistan; Tajikistan; Georgia [Caucasus]; Iran (EC-Iran, N-Iran, Iranian Aserbaijan); China (Gansu, Hebei, Heilongjiang, Henan, Jilin, Liaoning, Nei Mongol, Qinghai, Shaanxi, Shanxi); Mongolia; NW-India (Ladakh)	Whole herb, aerial parts	Sore throat; jaundice, liver heat, hepatitis; fever and cold; cough; stomach heat, stomach spasm, stomach bleeding, dysentery; food poisoning, green leg disease, clearing heat and drying dampness, cool blood and stopping bleeding, wound healing, headache, hematemesis	Zhu (1993) Jiang (2015) Morshedloo et al. (2020) Zhang et al. (2018)
*Dracocephalum nodulosum* Rupr	Kazakhstan; Uzbekistan; Kyrgyzstan; Tajikistan; China (Xinjiang); Mongolia	Whole herb	Expelling phlegm and relieving asthma; clearing heat and detoxification	Jiang (2015)
*D. nutans*	Siberia (Altai, Buryatia, Chita, Irkutsk, Krasnoyarsk, Tuva, W-Siberia, Yakutia); Russian Far East (Amur, Khabarovsk, Primorye); Kazakhstan; Kyrgyzstan; Tajikistan; Afghanistan (Kunar/Nuristan); China (Heilongjiang, Nei Mongol, Xinjiang); Mongolia; Pakistan (Chitral, Swat, Gilgit, Astor, Deosai, Baltistan); NW-India (Himachal Pradesh, Jammu & Kashmir, Dras, Zanskar)	Whole herb	Cough sputum panting, chronic bronchitis; eye swelling pain, hypertension; dizziness, tinnitus	Jiang (2015)
*D. origanoides*	Siberia (Altai, Tuva); Kazakhstan; Kyrgyzstan; Tajikistan; China (Xinjiang); Mongolia	Whole herb	Expelling phlegm and relieving asthma; clearing heat and detoxification	Jiang (2015)
*Dracocephalum palmatoides* C.Y.Wu and W.T.Wang	China (Xinjiang: Toli Shan)	Whole herb	Expelling phlegm and relieving asthma; clearing heat and detoxification	Jiang (2015)
*D. palmatum*	Siberia (Yakutia); Russian Far East (Kamchatka, Khabarovsk, Magadan)	Young shoots and flowers	Gastro-intestinal tract disorders and alcoholism	Olennikov et al. (2013) Olennikov et al. (2017)
*D. paulsenii*	Siberia (Tuva); Kazakhstan; Uzbekistan; Tajikistan; Afghanistan (Wakhan); China (Xinjiang); Mongolia; Pakistan (Chitral, Gilgit); NW-India (Jammu and Kashmir)	Stems, leaves, flowers	Relieving cough, relieving asthma and clearing heat	Jiang (2015)
*D. peregrinum*	Siberia (Altai, Irkutsk, Krasnoyarsk, Tuva, W-Siberia); Kazakhstan; China (Xinjiang); Mongolia	Whole herb	Clear heat and detoxicate; cooling blood and purging fire; relieving cough and asthma; clear phlegm	Yan (2020)
*D. rigidulum*	China (Nei Mongol)	Aerial part	Jaundice; sore throat; cold; cough; clearing heat and detoxification, stop bleeding, hematemesis, headache	Huang (2022)
*D. rupestre*	China (Hebei, Liaoning, Nei Mongol, Qinghai, Shanxi); North Korea	Whole herb, aerial part	Jaundice; sore throat; cold; cough; clearing heat and detoxification, stop bleeding, hematemesis, headache	Jiang (2015) Zhu (1993)
*D. ruyschiana*	Norway; Sweden; Germany (+Bayern, +Sachsen-Anhalt); Switzerland; Liechtenstein; Austria; Poland; Slovakia; Hungary; France; Italy; Croatia; Romania; Estonia; Latvia; Lithuania; Belarus; C-European Russia; E-European Russia; N-European Russia; NW-European Russia; Ukraine; Siberia (Altai, Buryatia, Chita, Irkutsk, Krasnoyarsk, Tuva, W-Siberia, Yakutia); Russian Far East (Amur); Kazakhstan; Turkmenistan; Uzbekistan; Kyrgyzstan; Northern Caucasus; Georgia [Caucasus]; Armenia; Turkey (NE-Anatolia); China (Heilongjiang, Nei Mongol, Xinjiang); Mongolia	Whole herb	Sore throat, laryngitis; jaundice, liver heat, hepatitis; cold; acute respiratory infection, cough; stomach heat, stomach spasm, stomach bleeding, gastric ulcers, dysentery, diarrhea; rheumatoid arthritis; food poisoning, green leg disease, clearing heat and drying dampness, cool blood and stopping bleeding, wound healing, headache	Jiang (2015) Zhu (1993)
*D. taliense*	China (Yunnan: Heqing, Dali)	Whole herb	Jaundice, hepatitis; adjusting the stomach	Jiang (2015) Deng et al. (2017)
*D. tanguticum*	China (Gansu, Qinghai, Sichuan: Maoxian, Songpan, Ma’erkang, Rangtang, Aba, Hongyuan, Ganzi, Kangding, Daofu, Luhuo, Ganzi, Dege, Shiqu, Seda, Xiangcheng, Daocheng, Derong, Muli); Tibet; Nepal; N-India (Chumbi)	Whole herb, aerial part, seedling	Chronic gastritis, gastric ulcer, epigastric pain; hepatitis, hepatomegaly; cough, sputum; ascites, edema	Jiang (2015) Guo et al. (2022)
*Dracocephalum thymiflorum* L	Slovakia; Romania; Bulgaria; S-European Russia; Belarus; C-European Russia; E-European Russia; N-European Russia; NW-European Russia; Ukraine; Siberia (Altai, Buryatia, Irkutsk, Krasnoyarsk, Tuva, W-Siberia); Kazakhstan; Northern Caucasus; Georgia [Caucasus]; Iran (EC-Iran, N-Iran)	Whole herb	Expelling phlegm and relieving asthma; clearing heat and detoxification	Jiang (2015)
*Hyssopus ambiguus* (Trautv.) Iljin ex Prochorov. and Lebel	Siberia (Altai, W-Siberia); Kazakhstan; Mongolia	Aerial part	Reducing swelling and relieving pain, clearing heat and detoxification	Jiang (2015)
*H. cuspidatus*	Siberia (Altai); Kazakhstan; China (Xinjiang); Mongolia	Whole herb	Relieve cough and asthma; clear phlegm; clear away the lung-heat, tracheitis, cough; cold and fever; tonsillitis; bladder and kidney stones; night sweats, relieve pain	Jiang (2015) Aihaiti et al. (2022) Jia et al. (2022)
*H. latilabiatus*	China (Xinjiang)	Whole herb	Heat-clearing, detoxification and anti-inflammation. For colds, fever, cough	Jiang (2015)
*H. officinalis*	Switzerland; Austria; Slovakia; Hungary; Spain; France; Italy; Montenegro; Serbia; Kosovo; North Macedonia; Albania; Bulgaria; C-European Russia; E-European Russia; Ukraine; Crimea; Siberia (Buryatia); Northern Caucasus; Georgia [Caucasus]; Armenia; Turkey (E-Anatolia, NE-Anatolia, SSW-Anatolia); Iran (EC-Iran, N-Iran, Iranian Aserbaijan); Pakistan (Baluchistan, Chitral, Swat); India (Himachal Pradesh, Jammu and Kashmir, Uttarakhand); Myanmar (Kachin, Sagaing)	Whole herb	Carminative and antispasmodic stomachic; gallstones; kidney stones; chronic bronchitis, asthma; tonsillitis; night sweats; rheumatic pains, bruises, wounds, anxiety, relaxation of muscles	Jiang (2015) Tahir et al. (2018) Semerdjieva et al. (2019)
*L. iberica*	Turkmenistan; Georgia [Caucasus]; Armenia; Turkey (E-Anatolia, Inner Anatolia, N-Anatolia, NE-Anatolia, S-Anatolia, SE-Anatolia, SE-Anatolia: Mesopotamian Anatolia, SSW-Anatolia, W-Anatolia); Iraq (NE-Iraq, NW-Iraq, SE-Iraq: Mesopotamia); Iran (EC-Iran, N-Iran, Iranian Aserbaijan, S-Iran, W-Iran); Lebanon (Antilebanon, C-Lebanon, coastal W-Lebanon); Syria (Jazira, NW-Syria, Jbel Druze, W-Syrian Mountains); Israel (N-Israel, Judean Desert); Jordania (S-Jordania, W-Jordania)	Seeds	Treating stress, fever, cough, nerve, liver, and kidney diseases	Al-Snafi (2019)
*L. royleana*	Siberia (W-Siberia); Kazakhstan; Turkmenistan; Uzbekistan; Kyrgyzstan; Tajikistan; Armenia; Iraq (S-Iraq, W-Iraq: Desert); Iran (EC-Iran, E-Iran, NE-Iran: Mts., N-Iran, Iranian Aserbaijan, S-Iran, W-Iran); Afghanistan (Baghlan, Balkh, Bamyan, Farah, Faryab, Herat, Kabul, Kandahar, Kunar/Nuristan, Laghman, Logar, Nangarhar, Paktia/Khost, Parwan, Zabul); Syria (C-Syrian Desert); Sinai peninsula (S-Sinai); Saudi Arabia (C-Saudi Arabia, N-Saudi Arabia, Midyan, Asir); Kuwait; China (Xinjiang); Pakistan (Chitral, Baluchistan, Kurram, Khyber, Hazara, Kohat, Pakistani Punjab, Rawalpindi, Swat, Baltistan, Skardu); India (Himachal Pradesh, Uttarakhand)	Seeds	Hepatic; renal diseases; sedation, treatment of various nervous	Ghannadi and Zolfaghari (2003) Jiang (2015)

**TABLE 3:** Pharmacological activities of the medicinal plants of genus *Dracocephalum* and its related genera.

NO	Activities	Species	Extract(s) or main metabolites	Types of study (*In vivo*/*In vitro*)	Dose	Key findings/Mode of action or biochemical and histopathological parameters studied	Reference
1	Hepatoprotective	*D. rupestre*	Phenylpropanoids: rosmarinic acid Flavonoids: eriodictyol	*In vivo* (mice) SOD, MDH, LDH, ALT, AST	50, 100, or 200 mg/kg	↓ALT ↓AST ↓MDH ↓LDH (Excl. 50 mg/kg)	Zhu et al. (2018)
		*D. heterophyllum*	-	*In vivo* (mice) *In vitro* (Kupffer cells)	20 mg/kg	↓Plasma ALT, AST, ↓IFN-γ, TNF-α	Zheng et al. (2016)
		*H. officinalis*	-	*In vitro* (FL83B mouse hepatocytes)	500 μg/mL	↑MTT cell viability	Chen et al. (2022a)
2	Anti-inflammatory	*D. komarovii*	Terpenoids: komarovin B, komarovin C, limonen-10-ol 10-*O*-*β*-D-glucopyranosyl- (1→2)-*β*-D-glucopyranoside	*In vitro* (RAW 264.7 cells)	1, 10, 50, 100 μM	↓NO	Toshmatov et al. (2019)
		*D. moldavica*	Terpenoids: Dracocephalumoid A-E Uncinatone Trichotomone F Caryopterisoid C	*In vitro* (RAW 264.7 cells)	1.12–5.84 μM	↓TNF-α, IL-1β ↓NO	Nie et al. (2021)
		*H. officinalis*	-	*In vivo* (Rats) *In vitro* (COX-1, COX-2)	50, 100, 200 mg/kg 5, 10, 20 μg/mL	↓COX-1 and COX-2 enzymes	Mićović et al. (2022)
		*H. cuspidatus*	Phenylpropanoid: Hyssopuside	*In vitro* (RAW 264.7 cells and mouse peritoneal macrophages)	10, 20, 40, 80 μM	↓NF-κB ↓TNF-α, IL-6, IL-1β ↓NO	Liu et al. (2021b)
3	Antimicrobial	*D. moldavica*	Essential oil Terpenoids: Geranyl acetate Geranial, Neral	*In vitro* (*Bacillus cereus*, *Escherichia coli*, *Listeria monocytogenes*, *Pseudomonas aeruginosa*, *Salmonella typhimurium*, and *Staphylococcus aureus*)	6.5 g/kg successive dilutions (100%–0.39%)	↓*E*. *coli* ↓*L*. *monocytogenes* ↓*S*. *typhimurium* ↓*S*. *aureus*	Aćimović et al. (2022)
		*H. officinalis*	Essential oil Terpenoids: isopiperitenone, pinocampheol, α-pinene	*In vitro* (*Fusarium graminearum* CCM F-683 and CCM 8244)	100, 500, 1,000 μg/mL	↓*F*. *graminearum* CCM F-683 and CCM 8244 growth (Excl. 100 μg/mL)	Harčárová et al. (2021)
		*H. cuspidatus*	Phenylpropanoids: caffeic acid, rosmarinic acid, oresbiusin A Flavonoids: salvigenin Others: daucosterol	*In vitro* (*Escherichia coli*, *Candida albicans* and *Staphylococcus aureus*)	-	↓*E*. *coli* ↓*C*. *albicans* ↓*S*. *aureus*	Shomirzoeva et al. (2020)
		*D. integrifolium*	Essential Oils Terpenoids: sabinene, eucalyptol	*In vitro* (*Bacillus subtilis*, *Pseudomonas aeruginosa*, *Escherichia coli*, *Saccharomyces cerevisiae*, and *Candida albicans*)	-	↓*B*. *subtilis* ↓*P*. *aeruginosa* ↓*E*. *coli* ↓*S*. *cerevisiae* ↓*C*. *albicans*	Zhou et al. (2019)
		*D. kotschyi*	Terpenoids: limonene, perilla aldehyde	*In vitro* (*Staphylococcus aureus* ATCC 25923 and *Escherichia coli* ATCC 25922)	-	↓*S*. *aureus* ↓*E*. *coli*	Moridi Farimani et al. (2017)
		*L*. *iberica*	Flavonoids: Rutin hydrate Phenylpropanoids:p-coumaric acid	*In vitro* (*Escherichia coli* ATCC-8739, *Staphylococcus aureus* ATCC-6538,*Pseudomonas aeruginosa* ATCC-9027 and *Enterobacter aerogenes* ATCC-13048)	0–100 mg/L	↓*P*. *aeruginosa* ↓*E*. *coli* ↓*S*. *aureus* ↓*E*. *aerogenes*	Yilmaz Kardas et al. (2023)
		*L*. *royleana*	-	*Staphylococcus aureus Enterobacter cloacae*, *Pseudomonas aeruginosa* and *Escherichia coli*	10,50,100 mg/mL	↓↓*S*. *aureus* ↓*E*. *cloacae* *P*. *aeruginosa* ↓*E*. *coli*	Mahmood et al. (2015)
4	Antihyperlipidemic	*D. kotschyii*	-	*In vivo* (Rats) *In vitro* (3T3-L1 cells)	0.25, 0.5 mL/rat 4 μL/mL	↓fasting blood glucose level, TC, TG, LDL; ↑HDL ↑p-FOXO1, p-AKT, PPARγ; ↓p-JNK, FOXO1, SREBP-1	Aslian and Yazdanparast (2018)
		*D. moldavica*	Total flavonoids	*In vivo* (Rats)	21 mg/kg, 42 mg/kg, 84 mg/kg	↓TG, LDLC ↑HDLC ↓ICAM-1, VCAM-1, PCNA (Excl. 21 mg/kg)	Quan et al. (2017)
		*L*. *royleana*	polysaccharide	*In vivo* (Rats)	200 mg/kg	↑GSH, Vitamin C, GPx, SOD ↓MDA, AOPP ↓CT, TG, LDL ↓AST, ALT, CK, Gamma GT, ALP, Urea, Creatinine, Uric acid	Mohammed et al. (2022)
5	Antitumor	*D. kotschyi*	Flavonoids: Calycopterin, Xanthomicrol	*In vivo* (mice)	20 mg/kg	↓Cell proliferation, VEGF activity	Zamani et al. (2016)
		*H. officinalis*	Total flavonoids, phenolic	*In vivo* (Rats) *In vitro* (C6 glioma cell)	100 mg/kg 50, 100, 200, 400, and 600 μg/mL	↑p53 and p21 mRNA; ↓SOD, CAT in tumor tissue ↓MTT cell viability (Excl. ≤100 mg/kg)	Khaksar et al. (2022)
		*D. taliense*	Flavonoid: 12-methoxy-18-hydroxy-sugiol, 2*α*,3*α*-dihydroxy-11*α*,12*α*-epoxy-urs-28,13*β*-olide	*In vitro* (HepG2 Cells, NCI-H1975)	0, 0.128, 0.256, 0.512, 1, 2, 5, 10, 20, 40, and 80 µM	-	Deng et al. (2017)
6	Anti-hyperglycemic	*D. tanguticum*	Phenylpropanoids: dratanguticumide B, dratanguticumide C Phenolic acids: dratanguticumide A	*In vitro* (3T3-L1 cells)	25 µM	↓glucose consumption rate	Ma et al. (2020)
	Improves myocardial ischemia	*D. moldavica*	Total flavonoids	*In vivo* (Rats)	2, 5, 12.5 μg/mL	↑LVDP, ±dp/dtmax, CF, HR, SOD, GSH/GSSG ↓CK, LDH, MDA	Jiang (2015)
7	Improve cerebral ischemia	*D. moldavica*	Total flavonoids	*In vivo* (Rats)	25, 50, 100 mg/kg	↓Brain tissue IL-6, IL-8, TNF-α ↑Brain tissue MDA, SOD, GSH-Px	Jia et al. (2017)
8	Improves memory impairment	*D. moldavica*	Phenylpropanoids: rosmarinic acid, oleanolic acid	*In vivo* (mice)	25, 50 and 100 mg/kg, p.o	↑ERK-CREB signaling cascade	Deepa et al. (2020)
9	Improves vascular dementia	*D. moldavica*	Total flavonoids Flavonoid: tilianin, luteolin, apigenin	*In vitro* (SH-SY5Y cells)	25, 50, 100 μg/mL	↑miR-3184–3p ↓miR-6875–5p	Liu et al. (2021a)
10	Antiviral activity	*D. foetidum D. nutans, D. fruticulosum*	Flavonoid: isosakuranetin glycosides; Phenylpropanoid oligomers	*In vitro* (Cells)	3.25–5.75 log10 TCID 50/mL	-	Sabrin et al. (2021)
11	Anti-allergic activity	*Dracocephalum argunense*	-	*In vivo* (mice) *In vitro* (HMC-1)	0.001–1 g/kg BW 0.001–1 mg/mL	↓TNF-α, ↓IL-6	Kim and Shin (2006)

## 7 Conclusion and prospects

In conclusion, we have shown that *Hyssopus*, *Lallemantia*, and *Dracocephalum* are closely related in many aspects, including molecular phylogeny, species distribution model, chemical composition, pharmacological activity, and traditional application. The division of these two genera into *Dracocephalum* was reasonable. Most flavonoids have flavonoid glycosides or flavonoid glycosides as their parent nuclear structure; therefore they have anti-inflammatory properties, but their pharmacological effects differ owing are different due to the different positions of the substituents. Modern pharmacological studies have justified some traditional medicinal uses, which may be partly attributed to the bioactivities of terpenoids, flavonoids, phenylpropanoids and phenolic acid, however, pharmacological mechanisms should be revealed to provide a scientific explanation for the traditional curative effect of *Dracocephalum* medicinal plants. In contrast, there are relatively few studies on the chemical metabolites of *Dracocephalum*, drug safety, effectiveness, and corresponding toxicological mechanisms, and the rationale for disease treatment remains uncertain. Although some species are used in traditional therapeutics, they lack verification of modern pharmacological research. Many metabolites have been identified using LC-MS, including indole alkaloids, flavonoids, terpenoids, and phenolic acids; however, some species of the genus *Dracocephalum* remain understudied with respect to their chemical metabolites. This review focused on *Sect. Sinodracon*, *Sect. Idiodracon*, *Sect. Calodracon*, and *Sect. Dracocephalum*. The commonly recorded efficacies of these sections are clearing away heat and toxic materials, relieving cough and asthma, eliminating phlegm, and protecting the liver. While studying the phylogenetic relationships of the genus *Dracocephalum*, we found that species in the *Hyssopus* and *Lallemantia* genera share a similar chemical composition, pharmacological activity, and traditional therapeutic effects. Network pharmacology has made some contributions to clarify the distance of plant genetic relationship from the perspective of mechanism.

In this review, the phamacophylogenetic relationship among *Hyssopus*, *Lallemantia*, and *Dracocephalum* is discussed from many standpoints, and on this basis, the related target pathways are predicted through network pharmacology, which helps to re-examine traditional usage, design/optimize experiments, to further unearth the associations among evolutionary relationship, chemical composition, and pharmacological activity. Based on previous studies, ecological adaptability evaluation was carried out to provide a research basis for the future cultivation of *Dracocephalum*, and clinical substitution of drugs among *Hyssopus*, *Lallemantia*, and *Dracocephalum*. Beyond this, there is considerable scope for future investigation into the conservation and utilization of plant resources, as well as the relationships of medicinal plants among these genera. For example, by employing species distribution prediction models in conjunction with various databases, it was possible to forecast potential suitable growth areas for endangered plants in these genera, followed by targeted conservation and cultivation efforts (Zhang et al., 2023). Furthermore, the application of genomics, transcriptomics, and metabolomics were crucial for exploring the origins of plant medicinal metabolism. Yang et al. (2023) sequenced the genomes of three Solanaceae species that produce hyoscyamine and scopolamine (HS) and one species that does not, revealing a shared biosynthetic pathway for HS across distantly related lineages, contributing to our understanding of HS biosynthesis. Correspondingly, the biosynthetic pathway of komaroviquinone, a distinctive pharmacological effect present from *D. kotschyi*, merits thorough investigation. This study not only enriches the theory of pharmacy but also provides a reference for the medicinal application of *Dracocephalum* medicinal plants. Unfortunately, for a long time, the medicinal value of the 60 species of *Dracocephalum* has not aroused enough attention or in-depth research; only a few species of chemical and pharmacological activities have been given due attention. For this reason, it is particularly urgent to find suitable substitutes and appropriate cultivation techniques. In this study, the genetic relationship between species of *Dracocephalum* was studied under the guidance of the genetic theory of medicinal plants, and it was skillfully transformed into the genetic relationship of modern scientific drugs, which was more helpful to understand and explore the medicinal value of *Dracocephalum*, and also provided the reference for the development of other medicinal plants. In addition, the internal relationships among the medicinal plants of *Dracocephalum* are still unclear, and these areas deserve further study in the future.
