# Supplementary figures and images for: Pharmacophylogenetic relationships of genus Dracocephalum and its related genera based on multifaceted analysis
Source: Front Pharmacol. 2024 Oct 3;15:1449426. doi: 10.3389/fphar.2024.1449426 (PMC11484080; doi:10.3389/fphar.2024.1449426)

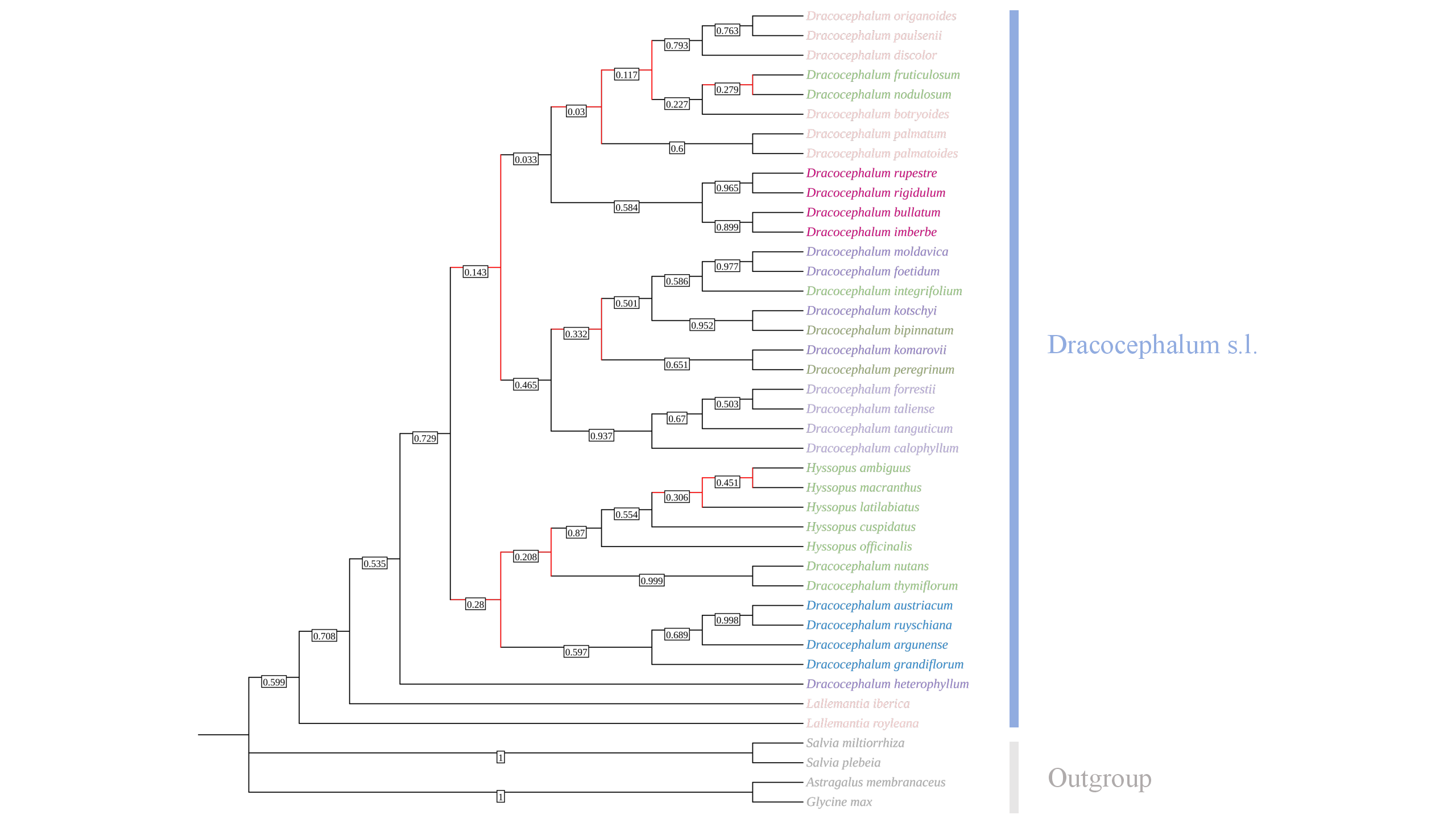

Supplement: Supplementary file 3 [file Image1.TIF]
