# Supplementary material for: Pharmacophylogenetic relationships of genus Dracocephalum and its related genera based on multifaceted analysis
Source: Front Pharmacol. 2024 Oct 3;15:1449426. doi: 10.3389/fphar.2024.1449426 (PMC11484080; doi:10.3389/fphar.2024.1449426)
Supplement: Supplementary file 7 [file DataSheet1.docx]

1. Aihaiti, K., Li, J., Yaermaimaiti, S., Liu, L., Xin, X., and Aisa, H.A. (2022). Non-volatile compounds of Hyssopus cuspidatus boriss and their antioxidant and antimicrobial activities. *Food Chemistry*(Apr.16), 374. doi:10.1016/j.foodchem.2021.131638
2. Aihaiti, K., Li, J., Xu, N.N., Tang, D., and Aisa, H.A. (2023). Monoterpenoid derivatives from Hyssopus cuspidatus Boriss. and their bioactivities. *Fitoterapia* 165, 105432. doi:10.1016/j.fitote.2023.105432
3. Ashrafi, B., Ramak, P., Ezatpour, B., and Talei, G.R. (2017). Investigation on chemical composition, antimicrobial, antioxidant, and cytotoxic properties of essential oil from Dracocephalum kotschyi Boiss. *African journal of traditional, complementary, and alternative medicines : AJTCAM* 14(3), 209–217. doi:10.21010/ajtcam.v14i3.23
4. Dai, L.M., Zhao, C.C., Jin, H.Z., Tang, J., Shen, Y.H., Li, H.L., et al. (2008). A new ferulic acid ester and other constituents from Dracocephalum peregrinum. *Archives of pharmacal research* 31(10), 1325–1329. doi:10.1007/s12272-001-2113-2
5. Dehaghi, N.K., Lai, D., Amanzadeh, Y., Sadat-Ebrahimi, S.S., and Proksch, P. (2012). A new putrescine bisamide phenolic glycoside from the seeds of lallemantia iberica (m. bieb.) fisch. & c. a. mey. *Phytochemistry Letters* 5(3), 643-646. doi:10.1016/j.phytol.2012.06.013
6. Deng, Y., Hua, J., Wang, W., Zhan, Z., Wang, A., and Luo, S. (2017). Cytotoxic Terpenoids from the Roots of Dracocephalum taliense. *Molecules (Basel, Switzerland)* 23(1). doi:10.3390/molecules23010057.
7. Fathiazad, F., Mazandarani, M., and Hamedeyazdan, S. (2011). Phytochemical analysis and antioxidant activity of Hyssopus officinalis L. from Iran. *Advanced pharmaceutical bulletin* 1(2), 63–67. doi:10.5681/apb.2011.009
8. Fattahi, A., Shakeri, A., Tayarani-Najaran, Z., Kharbach, M., Segers, K., Heyden, Y.V., et al. (2021). UPLC-PDA-ESI-QTOF-MS/MS and GC-MS analysis of Iranian Dracocephalum moldavica L. *Food science & nutrition* 9(8), 4278–4286. doi:10.1002/fsn3.2396
9. Fattahi, M., Nazeri, V., Torras-Claveria, L., Sefidkon, F., Cusido, R. M., Zamani, Z., and Palazon, J. (2013). Identification and quantification of leaf surface flavonoids in wild-growing populations of Dracocephalum kotschyi by LC-DAD-ESI-MS. *Food chemistry* 141(1), 139–146. doi:10.1016/j.foodchem.2013.03.019
10. Ferreira, E.deO., Fernandes, M.Y., Lima, N.M., Neves, K.R., Carmo, M.R., Lima, F.A., et al. (2016). Neuroinflammatory response to experimental stroke is inhibited by eriodictyol. *Behavioural brain research* 312, 321–332.doi:10.1016/j.bbr.2016.06.046
11. Fu F., (2009). Studies on the chemical constituents of Polytrichum commune, Conoscyphus trapezioides and Dracocephalum peregrinum. [Doctoral dissertation], Second Military Medical University.
12. Fu, P., Zhao, C.C., Tang, J., Shen, Y.H., Xu, X.K., and Zhang, W.D. (2009). New flavonoid glycosides and cyanogenic glycosides from Dracocephalum peregrinum. *Chemical & pharmaceutical bulletin* 57(2), 207–210. doi:10.1248/cpb.57.207
13. Gao, J., Wang, Z., Chen, D., Peng, J., Xie, D., Lin, Z., et al. (2022). Metabolomic characterization of the chemical compositions of Dracocephalum rupestre Hance. *Food research international (Ottawa, Ont.)* 161, 111871. doi:10.1016/j.foodres.2022.111871
14. Ghannadi, A., and Zolfaghari, B. (2010). Compositional analysis of the essential oil of lallemantia royleana (benth. in wall.) benth. from iran. *Flavour & Fragrance Journal* 18(3), 237-239. doi:10.1002/ffj.1215
15. Ghavam, M., Manconi, M., Manca, M.L., and Bacchetta, G. (2021). Extraction of essential oil from Dracocephalum kotschyi Boiss. (Lamiaceae), identification of two active compounds and evaluation of the antimicrobial properties. *Journal of ethnopharmacology* 267, 113513. doi:10.1016/j.jep.2020.113513
16. Ghavam M. (2022). A GC-MC analysis of chemical compounds and identification of the antibacterial characteristics of the essential oil of two species exclusive to Iranian habitats: New chemotypes. *PloS one* 17(10), e0273987. doi:10.1371/journal.pone.0273987
17. Guerrini, A., Sacchetti, G., Echeverria Guevara, M.P., Paganetto, G., Grandini, A., Maresca, I., et al. (2021). Wild Italian Hyssopus officinalis subsp. Aristatus (Godr.) Nyman: From Morphological and Phytochemical Evidences to Biological Activities. *Plants (Basel, Switzerland)* 10(4), 631. doi:10.3390/plants10040631
18. Haitz, F., Radloff, S., Rupp, S., Fröhling, M., Hirth, T., and Zibek, S. (2018). Chemo-Enzymatic Epoxidation of Lallemantia IbericaSeed Oil: Process Development and Economic-Ecological Evaluation. *Applied biochemistry and biotechnology* 185(1), 13–33. doi:10.1007/s12010-017-2630-1
19. Hu, X., Mola, Y., Su, W.L., Wang, Y., Zheng, R.F., and Xing, J.G. (2023). A network pharmacology approach to decipher the total flavonoid extract of Dracocephalum Moldavica L. in the treatment of cerebral ischemia- reperfusion injury. *PloS one* 18(7), e0289118. doi:10.1371/journal.pone.0289118
20. Ilkei, V.; Spaits, A.; Prechl, A. (2016). Biomimetic synthesis and HPLC-ECD analysis of the isomers of dracocephins A and B. *Beilstein J Org Chem*. 12:2523-2534. doi:10.3762/bjoc.12.247.
21. Javad, S.R., Seyedeh Mahsan, H.A., Majid, S.R., and William, N.S. (2015). Chemical composition, antifungal and antibacterial activities of essential oil from lallemantia royleana (benth. in wall.) benth. *Journal of Food Safety*. doi:10.1111/jfs.12139
22. Kakasy, A.Z. , É. Lemberkovics, B. Simándi, Lelik, L., and É. Szöke. (2006). Comparative study of traditional essential oil and supercritical fluid extracts of moldavian dragonhead (dracocephalum moldavica l.). *Flavour and Fragrance Journal* 21(4), 598-603. doi:10.1002/ffj.1569
23. Kashchenko, N.I., Jafarova, G.S., Isaev, J.I., Olennikov, D.N., and Chirikova, N.K. (2022). Caucasian Dragonheads: Phenolic Compounds, Polysaccharides, and Bioactivity of Dracocephalum austriacum and Dracocephalum botryoides. *Plants-Basel* 11(16). doi: 10.3390/plants11162126.
24. Lee, S. B., Cha, K. H., Kim, S. N., Altantsetseg, S., Shatar, S., Sarangerel, O., et al. (2007). The antimicrobial activity of essential oil from Dracocephalum foetidum against pathogenic microorganisms. J. Microbiol. (Seoul, Korea) 45 (1), 53–57. Available at: https://pubmed.ncbi.nlm.nih.gov/17342056/.
25. Li, G.P., Zhao, J.F., Yang, L.J., Yang, X.D., and Li, L. (2006). Three new triterpenoids from Dracocephalum forrestii. *Helvetica Chimica Acta* 89(12), 3018-3022. doi:10.1002/hlca.200690271
26. Li, G.P., Zhao, J.F., Yang, H.Y., Yang, X.D., Zhang, H.B., and Li, L. (2007). Arylglycerol glucosides from Dracocephalum forrestii. *Journal of Asian natural products research* 9(3-5), 457–461. [doi:](https://doi:)10.1080/10286020600652624
27. Li, S.M., Yang, X.W., Li, Y.L., Shen, Y.H., Feng, L., Wang, Y.H., et al. (2009). Chemical constituents of Dracocephalum forrestii. Planta medica 75(15), 1591–1596. doi:10.1055/s-0029-1185868
28. Liu, G., Xu, Z., Chen, J., Lang, G., Tian, Q., Shen, Y., et al. (2009). On-line strategies for the identification of unknown flavone glycosides in Dracocephalum tanguticum Maxim. Journal of chromatography. *B, Analytical technologies in the biomedical and life sciences* 877(24), 2545–2550. doi:10.1016/j.jchromb.2009.06.040
29. Ma, E.G., Wu, H.Y., Hu, L.J., Wei, M., Mou, L.Y., and Li, G.P. (2020). Three new phenylacetamide glycosides from Dracocephalum tanguticum Maxim and their anti-hyperglycemic activity. *Natural product research* 34(13), 1827–1835. doi:10.1080/14786419.2018.1562451
30. Misra, L.N., and Ahmad, A. (1992). An Oxygenated Tetrahydrobergamotene from the Essential Oil of Dracocephalum nutans*. *Planta medica* 58(5), 478–479. doi:10.1055/s-2006-961524
31. Misra, L.N., Shawl, A.S., and Raina, V.K. (1988). Volatile Constituents of Dracocephalum nutans. *Planta medica* 54(2), 165–166. doi:10.1055/s-2006-962380
32. Moridi Farimani, M., Mirzania, F., Sonboli, A., and Moghaddam, F.M. (2017). Chemical composition and antibacterial activity of Dracocephalum kotschyi essential oil obtained by microwave extraction and hydrodistillation. *International Journal of Food Properties* 20(sup1), 306–315. doi:10.1080/10942912.2017.1295987
33. Nie, L., Li, R., Huang, J., Wang, L., Ma, M., Huang, C. et al. (2021). Abietane diterpenoids from Dracocephalum moldavica L. and their anti-inflammatory activities in vitro. *Phytochemistry*, 184, 112680. doi:10.1016/j.phytochem.2021.11268
34. Nori-Shargh, D., Kiaei, S.M., Deyhimi, F., Mozaffarian, V., and Yahyaei, H. (2009). The volatile constituents analysis of Lallemantia iberica (M.B.) Fischer & Meyer from Iran. *Natural product research* 23(6), 546–548. doi:10.1080/14786410601132394
35. Numonov, S.R., Qureshi, M.N., and Aisa, H.A. (2015). Development of HPLC Protocol and Simultaneous Quantification of Four Free Flavonoids from Dracocephalum heterophyllum Benth. *International journal of analytical chemistry*, 2015, 503139. doi:10.1155/2015/503139
36. Olennikov, D.N., Chirikova, N.K., Okhlopkova, Z.M., and Zulfugarov, I.S. (2013). Chemical composition and antioxidant activity of Tánara Ótó (Dracocephalum palmatum Stephan), a medicinal plant used by the North-Yakutian nomads. *Molecules* 18(11), 14105-14121. doi:10.3390/molecules181114105.
37. Saeidnia, S., Gohari, A.R., Uchiyama, N., Ito, M., Honda, G., and Kiuchi, F. (2004). Two new monoterpene glycosides and trypanocidal terpenoids from Dracocephalum kotschyi. *Chemical & pharmaceutical bulletin* 52(10), 1249–1250. doi:10.1248/cpb.52.1249
38. Selenge, E., Murata, T., Kobayashi, K., Batkhuu, J., and Yoshizaki, F. (2013). Flavone tetraglycosides and benzyl alcohol glycosides from the Mongolian medicinal plant Dracocephalum ruyschiana. *Journal of natural products* 76(2), 186–193. doi:10.1021/np300609u
39. Selenge, E., Murata, T., Tanaka, S., Sasaki, K., Batkhuu, J., and Yoshizaki, F. (2014). Monoterpene glycosides, phenylpropanoids, and acacetin glycosides from Dracocephalum foetidum. *Phytochemistry* 101, 91–100. doi:10.1016/j.phytochem.2014.02.007
40. Shi, Q.Q., Zhao, J.Q., Dang, J., Yuan, X., and Wang, Q.L. (2018). Triterpenes, flavonoids, and lignans from Dracocephalum heterophyllum. *Chemistry of Natural Compounds* 54(5), 970-972. doi:10.1007/s10600-018-2524-7
41. Shomirzoeva, O., Li, J., Numonov, S., Atolikshoeva, S., and Aisa, H.A. (2020). Chemical components of Hyssopus cuspidatus Boriss.: isolation and identification, characterization by HPLC-DAD-ESI-HRMS/MS, antioxidant activity and antimicrobial activity. *Natural product research* 34(4), 534–540. doi:10.1080/14786419.2018.1488710
42. Singh, N., Kaul, V.K., Megeji, N.W., Singh, V., and Ahuja, P.S. (2008). Essential oil composition of three accessions of Dracocephalum heterophyllum Benth. cultivated at Palampur, India. *Natural product research* 22(11), 927–936. doi:10.1080/14786410701642847
43. Stappen, I., Wanner, J., Tabanca, N., Wedge, D.E., Ali, A., Kaul, V.K., et al. (2015). Chemical composition and biological activity of essential oils of Dracocephalum heterophyllum and Hyssopus officinalis from Western Himalaya. *Natural product communications* 10(1), 133–138. doi:10.1177/1934578X1501000131
44. Suleimen, E.M., Tursynova, N.K., Ibataev, Z.A., Iskakova, Z.B., and Ishmuratova, M.Y. (2015). Constituent composition and biological activity of essential oil from Hyssopus ambiguus. *Chemistry of Natural Compounds* 51(6), 1186-1187. doi:10.1007/s10600-015-1527-x
45. Suleimen, E.M. , Myrzagalieva, A.B. , Ibataev, Z.A. , Iskakova, Z.B. , Samarkhanov, T.N. , and Medeubaeva, B.Z. (2017). Constituent composition and biological activity of essential oil from dracocephalum peregrinum. *Chemistry of Natural Compounds* 53(1), 173-174. doi:10.1007/s10600-017-1941-3
46. Uchiyama, N., Kiuchi, F., Ito, M., Honda, G., Takeda, Y., Khodzhimatov, O. K., et al. (2003). New icetexane and 20 norabietane diterpenes with trypanocidal activity from Dracocephalum komarovi. *Journal of natural products* 66(1), 128–131. doi:10.1021/np020308z
47. Uchiyama, N., Kiuchi, F. Ito, M., Honda, G., and Ashurmetov, O.A. (2006). Trypanocidal constituents of Dracocephalum komarovi. *Tetrahedron* 62(18), 4355-4359. doi:10.1016/j.tet.2006.02.067
48. Wang, J.M., Sun, J.F., Jin, L., Wang, M.J., Huang, Y.Y., Jin, M., et al. (2022). One novel naphthalene derivative and other constituents with anti-complementary activities from the aerial parts of Dracocephalum moldavica. *Journal of Asian natural products research* 24(12), 1177–1184. doi:10.1080/10286020.2021.2024518
49. Wang, J., Sun, J., Jin, L., Wang, M., Huang, Y., Jin, M., Zhou, W., and Li, G. (2023a). A new monoterpenoid glycoside and a new phenolic glycoside isolated from Dracocephalum moldavica and their anti-complementary activity. *Natural product research* 37(2), 169–179. doi:10.1080/14786419.2021.1957885
50. Wang, J., Sun, J., Jin, L., Huang, Y., Wang, M., Jin, M., et al. (2023b). Four new terpenoids and other metabolites with potential anti-complementary activities from the aerial parts of Dracocephalum moldavica (Lamiaceae). *Natural product research* 37(13), 2135–2143. doi:10.1080/14786419.2022.2030329
51. Wang, L., Wang, S., Yang, S., Guo, X., Lou, H., and Ren, D. (2012). Phenolic alkaloids from the aerial parts of Dracocephalum heterophyllum. *Phytochemistry* 82, 166–171. doi:10.1016/j.phytochem.2012.06.021.
52. Wang, S.Q., Ren, D.M., Xiang, F., Wang, X.N., Zhu, C.J., Yuan, H.Q., et al. (2009). Dracotanosides A-D, spermidine glycosides from Dracocephalum tanguticum: structure and amide rotational barrier. *Journal of natural products* 72(6), 1006–1010. doi:10.1021/np900140s
53. Wang, N., and Yang, X.W. (2010). Two new flavonoid glycosides from the whole herbs of Hyssopus officinalis. *Journal of Asian natural products research* 12(12), 1044–1050. doi:10.1080/10286020.2010.533120
54. Yan Y.L. (2020).Chemical Constituents Extraction and Antitumor Activity of Dracocephalum peregrinum Linn. [Doctoral dissertation], Northeast Normal University. doi:10.27011/d.cnki.gdbsu.2020.001747.
55. Zeng, Q., Chang, R., Qin, J., Cheng, X., Zhang, W., and Jin, H. (2011). New glycosides from Dracocephalum tanguticum maxim. *Archives of pharmacal research* 34(12), 2015–2020. doi: 10.1007/s12272-011-1202-0
56. Zhang, C., Li, H., Yun, T., Fu, Y., Liu, C., Gong, B., and Neng, B. (2008). Chemical composition, antimicrobial and antioxidant activities of the essential oil of Tibetan herbal medicine Dracocephalum heterophyllum Benth. *Natural product research* 22(1), 1–11. doi:10.1080/14786410701619076
57. Zhang, G.Y., Ling J.Y., and Cui Z.J. (2007). Supercritical CO2 extraction of essential oil from Dracocephalum tanguticum Max‐im and analysis by GC‐MS. *Journal of Liquid Chromatography and Related Technologies* 30(2), 287-292.doi:10.1080/10826070601064607
58. Zhang, H., Wang, S., Liu, Q., Zheng, H., Liu, X., Wang, X., et al. (2021). Dracomolphin A-E, new lignans from Dracocephalum moldavica. *Fitoterapia* 150, 104841. doi:10.1016/j.fitote.2021.104841
59. Zhou, S., Wei, C., Zhang, C., Han, C., Kuchkarova, N., and Shao, H. (2019). Chemical Composition, Phytotoxic, Antimicrobial and Insecticidal Activity of the Essential Oils of Dracocephalum integrifolium. *Toxins* 11(10), 598. doi:10.3390/toxins11100598
60. Zhou, X., Hai-Yan, G., Tun-Hai, X., and Tian, S. (2010). Physicochemical evaluation and essential oil composition analysis of Hyssopus cuspidatus Boriss from Xinjiang, China. *Pharmacognosy magazine* 6(24), 278–281. doi:10.4103/0973-1296.71790
